# Aqueous Na_2_WO_4_/H_2_O_2_: an efficient tunable catalytic medium for selective oxidation of β-naphthol to diverse products

**DOI:** 10.1039/d6ra03782h

**Published:** 2026-07-06

**Authors:** Mohammad M. Mojtahedi, Elham Ashoori, M. Saeed Abaee

**Affiliations:** a Organic Chemistry Department, Chemistry and Chemical Engineering Research Center of Iran P.O. Box 14335-186 Tehran Iran mojtahedi@ccerci.ac.ir

## Abstract

The aqueous-phase oxidation of β-naphthol 1 with hydrogen peroxide in the presence of sodium tungstate dihydrate (Na_2_WO_4_) and various basic or acidic reagents demonstrated remarkable condition-dependent product selectivity. By simple adjustment of the conditions, the transformation of 1 would be directed towards one of the products of choice 2–6 in high yields. In the presence of NaOH, Lawsone 2 was obtained quickly and efficiently. In contrast, use of a mild acidic reagent (acetic acid) afforded 1,1′-bi-2-naphthol 3, selectively. Alternatively, higher concentrations of acetic acid promoted an oxidation–dimerization–cyclization process to give benzochromene 4. By the replacement of AcOH with formic acid or ClCH_2_CO_2_H, the selectivity of the process shifted towards the high yield formation of 5, and 6, respectively. The results emphasized that the pivotal role of aqueous media and the relative pH of the reaction provided access to high yields of structurally diverse products in short reaction times. At the same time, the operations were quite simple and capable of being processed at higher scales, as demonstrated for ten-gram synthesis of Lawsone 2. In all reactions, the catalyst could be successfully recycled and reused efficiently in the next reactions, while products are obtained directly without the need for cumbersome and costly chromatographic operations.

## Abstract 2

An aqueous Na_2_WO_4_/H_2_O_2_ solution provides a tuneable catalytic medium for selective and efficient oxidation of β-naphthol to five different products, depending on the conditions and the pH of the reaction.

## Graphical abstract

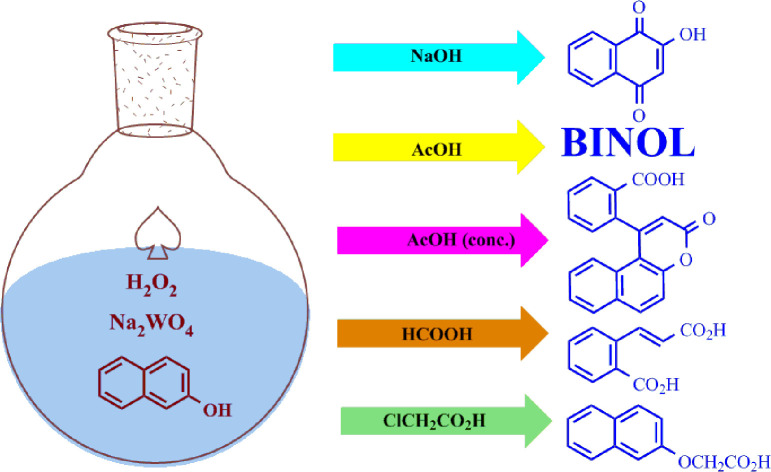

## Introduction

**Fig. 1:**
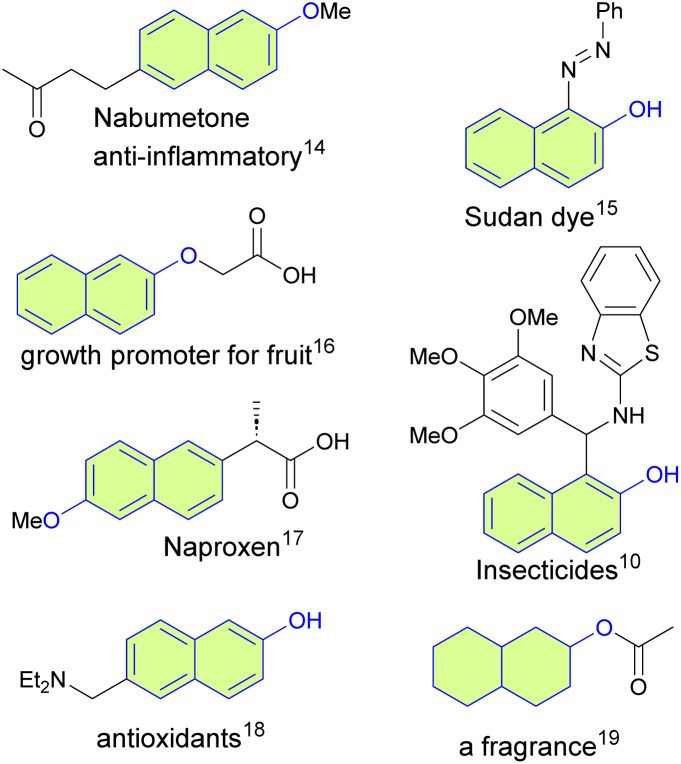
Examples of useful compounds containing the β-naphthol fragment.^14–19^

β-Naphthol derivatives constitute a very important category of fused bicyclic compounds, widely occurring in the structures of numerous natural and synthetic products. These compounds behave as biochemical and medicinal active candidates, like anticancer,^1^ antiviral,^2^ antimalarial,^3^ antibacterial,^4^ anti-hepatitis,^5^ anti-HIV,^6^ and anti-inflammatory^7^ agents. β-Naphthols are also extensively used in synthetic organic chemistry for the preparation of pigments,^8^ dyes,^9^ pesticides,^10^ antifungals,^11^ antiseptics,^12^ and antioxidants.^13^ Some illustrative structures of important synthetic and naturally occurring molecules containing the β-naphthol backbone are exemplified in Fig. 1.

**Fig. 2:**
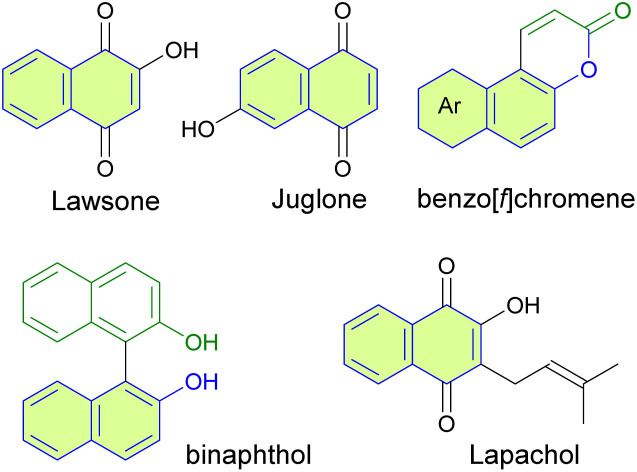
Important β-naphthol-derived structures.

Due to the importance of β-naphthol derivatives, the chemistry of these compounds including their dimerization,^20^ dearomatization,^21^ oxidation,^22^ homo-coupling,^23^ heterocoupling,^24^ and hydroarylation^25^ reactions are important and investigated extensively in recent decades.^26,27^ On the other hand, some of the naphthol containing structures are associated with environmental concerns, and new methods are developed in recent years for their detection and measurement.^28^ Among the important derivatives of β-naphthol are Lawsone, Juglone, and lapachol, which are naturally occurring products.^29^ Other related important motifs are bis-naphthols and benzochromenes, acting as catalysts^30^ and biologically active structures,^31^ respectively (Fig. 2). These valuable compounds are the result of oxidative dearomatization,^32^ dimerization,^33^ and cleavage-lactonization^34^ reactions, respectively.

**Scheme 1:**
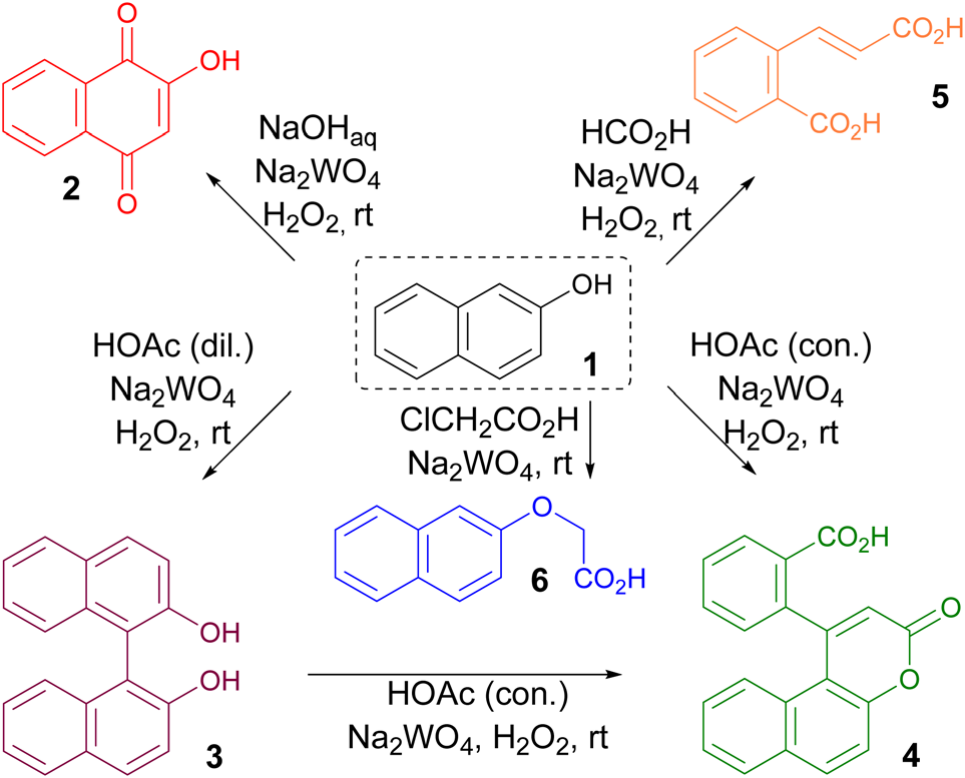
Reaction routes to various products.

We are interested in developing various environmentally sustainable procedures in synthetic organic chemistry.^35^ In this context, we have conducted sonochemical,^36^ aqueous conditioned,^37^ and heterogeneous catalyzed^38^ methods in various functional group transformations. On this track, we were persuaded to reinvestigate into the oxidation reactions of β-naphthol under different conditions, so that we would develop useful procedures to access value-added β-naphthol-based materials *via* straightforward procedures. As a result, we report herein the reactions of 1 under aqueous Na_2_WO_4_ catalysis, where convenient preparation of 2–6 is accomplished (Scheme 1). Reactions proceed efficiently, and the catalyst is recycled several times conveniently.

## Results and discussion

Our experimental observations showed that the room temperature oxidation of β-naphthol under basic or acidic conditions proceeds differently, depending on the chosen reagent and conditions, as summarized in Table 1.

**Table 1:** Products synthesis optimization

Entry	Additive (mol%)	Catalyst^a^	Solvent	Time	Product	Yield^b^ (%)
1	NaOH (40)	Na_2_WO_4_·2H_2_O	H_2_O	45 min	2	92
2	NaOH (40)	—	H_2_O	180 min	2	50
3	NaOH (25)	Na_2_WO_4_·2H_2_O	H_2_O	45 min	2	70
4	NaOH (65)	Na_2_WO_4_·2H_2_O	H_2_O	45 min	2	65
5	NaOH (40)	Na_2_WO_4_·2H_2_O	EtOH	60 min	2	60
6	NaOH (40)	Na_2_WO_4_·2H_2_O	EtOH/H_2_O	45 min	2	75
7	NaOH (40)	Na_2_WO_4_·2H_2_O	PhMe	45 min	2	40
8	NaOH (40)	Na_2_WO_4_·2H_2_O	CH_2_Cl_2_	45 min	2	30
9	NaOH (40)	ZrOCl_2_·8H_2_O	H_2_O	45 min	2	80
10	NaOH (40)	V_2_O_5_	H_2_O	45 min	2	75
11	KOH (40)	Na_2_WO_4_·2H_2_O	H_2_O	60 min	2	65
12	K_2_CO_3_ (40)	Na_2_WO_4_·2H_2_O	H_2_O	60 min	2	45
13	AcOH (10)	Na_2_WO_4_·2H_2_O	H_2_O	15 min	3	93
14	AcOH (40)	Na_2_WO_4_·2H_2_O	H_2_O	30 min	4	95
15	AcOH (40)	V_2_O_5_	H_2_O	30 min	4	50
16	AcOH (40)	Na_2_MoO_4_·2H_2_O	H_2_O	30 min	4	30
17	PhCO_2_H (40)	Na_2_WO_4_·2H_2_O	H_2_O	45 min	4	0
18	Oxalic acid (40)	Na_2_WO_4_·2H_2_O	H_2_O	45 min	4	0
19	TsOH (40)	Na_2_WO_4_·2H_2_O	H_2_O	45 min	4	0
20	Hexanoic acid (40)	Na_2_WO_4_·2H_2_O	H_2_O	45 min	4	10
21	HCO_2_H (10)	Na_2_WO_4_·2H_2_O	H_2_O	20 min	5	94
22	HCO_2_H (40)	Na_2_WO_4_·2H_2_O	H_2_O	30 min	—	—
23	2-ClCH_2_CO_2_H (100)	Na_2_WO_4_·2H_2_O	H_2_O	45 min	6	92
24	NaOH^c^	Na_2_WO_4_·2H_2_O	EtOH	4 h	2	32
25	NaOH^c^	Na_2_WO_4_·2H_2_O	PhMe	4 h	2	11

a The catalyst amount in all reactions is 1.5 mol%.

b Isolated yields.

c Saturated in EtOH.

Treatment of 1 with H_2_O_2_ in an aqueous NaOH (40 mol%) mixture and in the presence of Na_2_WO_4_ produced 92% Lawsone 2 after 45 min (entry 1). In the absence of the catalyst, the process only gave 50% of 2 within 3 h (entry 2), conveying the positive effect of the catalyst on the progress of the reaction. Variation in the amount of the base (entries 3–4) or the type of the solvent (entries 5–8) did not improve the results. Similarly, change of the catalyst (entries 9–10) or the base (entries 11–12) did not change the outcome significantly. Interestingly, when the conditions were switched by using an acid to replace NaOH, the product of the process was altered completely. Thus, in the presence of lower concentrations of AcOH, H_2_O_2_ oxidation of β-naphthol produced 93% of binol ([1,1′-binaphthalene]-2,2′-diol) 3 after 15 min mixing at rt (entry 13). When this reaction was conducted in the presence of radical scavengers (TEMPO^39^ and *t*-BuOH^40^), formation of no product 3 was observed. In contrast, with higher amounts of AcOH, the tetracyclic product 4 was obtained in 95% yield and within only 30 min (entry 14). Again, the use of other catalysts (entries 15–16) or acids (entries 17–20) did not improve the results. The exception was when formic acid was used in low concentrations, giving 5 in high yields (entry 21). In contrast, with higher quantities of the acid, the reaction proceeded harshly and relatively out of control, perhaps due to higher activity of the acid (entry 22). Alternatively, the use of ClCH_2_CO_2_H as a substitute for AcOH gave the alkylated product 6 (entry 23). This occurred prior to the addition of H_2_O_2_ and no further change was noticed by the addition of H_2_O_2_.

To clarify the role of water in the progress of the reactions, two additional experiments were conducted in which non-aqueous alkaline media were used (entries 24–25). As a result, the respective product was formed within much longer times and in substantially lower yields. These results supported that water or another protic solvent (EtOH) is pivotal for the progress of the reactions, since in toluene (an aprotic solvent) mixtures, the yields dropped dramatically.

**Fig. 3:**
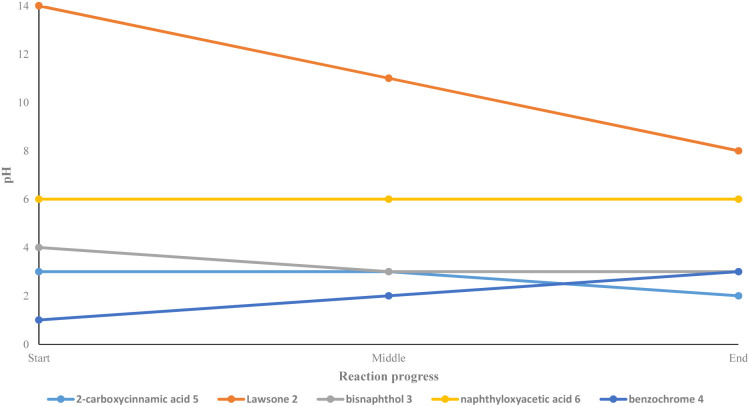
The pH variation during the course of the reactions.

The pH variation during the reaction was recorded at the start, middle, and end stages for all products, as graphically depicted in Fig. 3. Lawsone 2 showed a pronounced decrease in pH, whereas in the synthesis of naphthyloxyacetic acid 6, the pH remained nearly unchanged throughout the reaction. Alternatively, negligible pH variations were observed during the preparation of binaphthol 3 and 2-carboxycinnamic acid 5. At the same time, benzochromene 4 exhibited a gradual increase in pH, reflecting distinct acid–base behaviors of the investigated substrates. Regarding the acid strength, it is demonstrated in some other H_2_O_2_-promoted oxidative reactions that there is no linear relationship between the p*K*_a_ of the acids and the conversion rates of the oxidation reactions, as for example concluded by Reedijk *et al.*^41^ Tungstate speciation in aqueous solutions at different pH values leading to different synthetic pathways is well documented.^42^

**Fig. 4:**
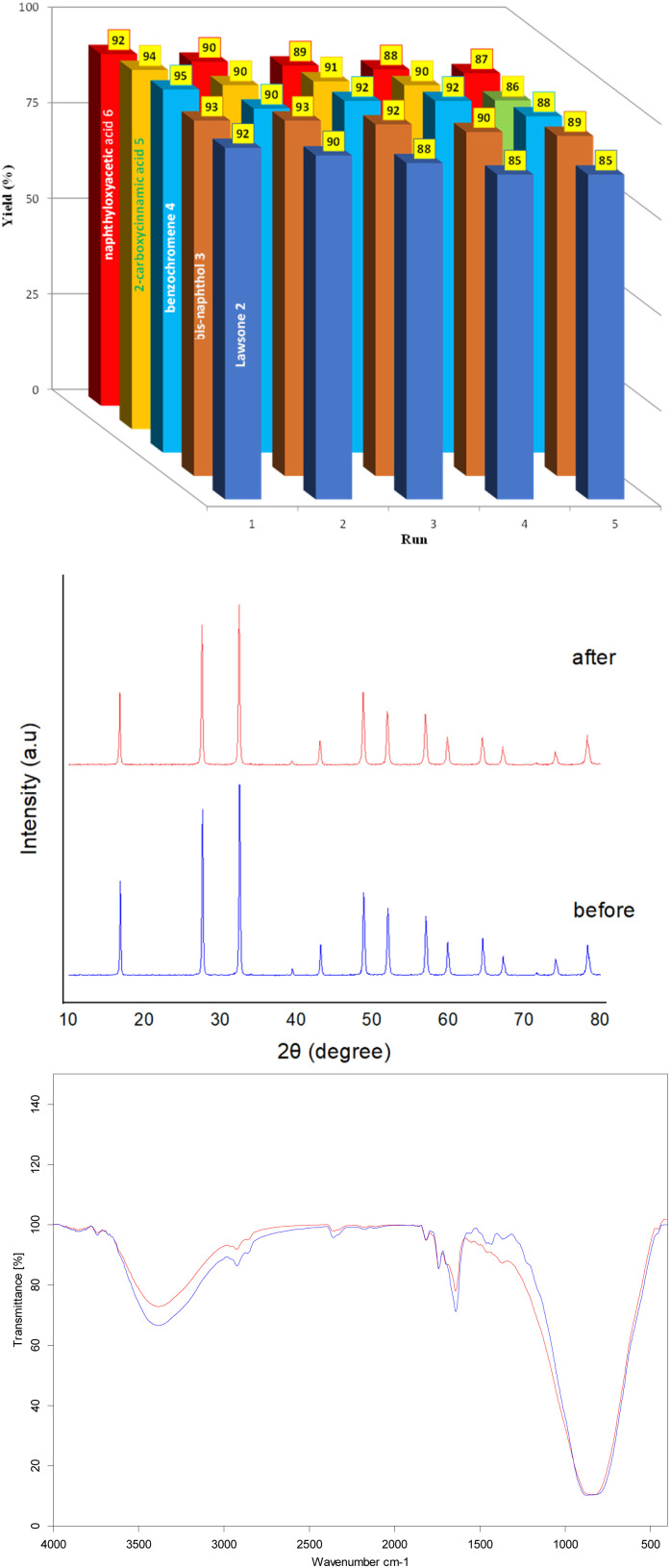
Recovery of the catalyst (top) for the reactions. XRD pattern (middle) and FTIR spectra (bottom) of the catalyst before (blue) and after (red) recycling in the synthesis of 2.

From economic and green chemistry points of view, this is pivotal to recover and reuse the reagents and catalysts to reduce the release of chemicals into the environment and lower the financial costs. Therefore, next we could recover the catalyst by hot EtOH filtration, and after makeup, reuse it successfully in the next reactions. This was executed for all five sets of optimum conditions for the synthesis of products 2–6. The results are summarized in Fig. 4 (top) for five consecutive uses of the catalyst in each reaction. Moreover, to verify the composition of the catalyst after recycling runs, the recovered solid was dried in an oven (80 °C), and then subjected to XRD and FTIR analyses. Fig. 4-middle and Fig. 4-bottom illustrate the spectra of the fresh and the recovered catalyst, which are nearly superimposable for both sets of experiments. In addition, we evaluated the green chemistry efficiency for the transformation of β-naphthol to the products using atom economy (AE), reaction mass efficiency (RME), and *E*-factor. The evaluation showed that all reactions proceeded with high yields (92–95%) and favorable metrics (AE ≈ 72–99%, RME ≈ 68–93%, *E*-factor ≈ 0.08–0.40), indicating low waste generation and good compliance with sustainable and environmentally friendly practices.

**Fig. 5:**
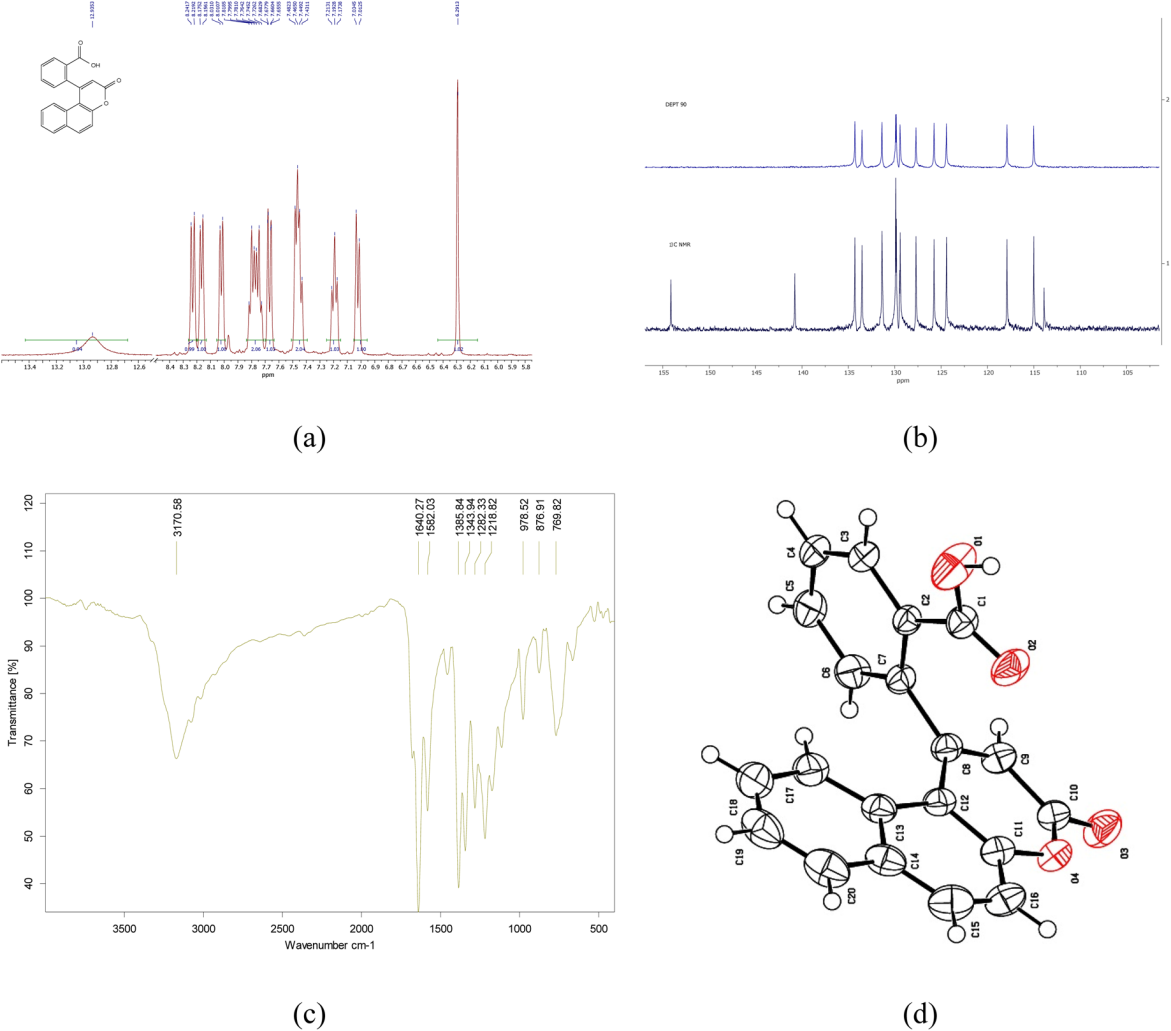
(a) ^1^H NMR, (b) ^13^C NMR (DEPT-90 and decoupled), and (c) FTIR spectra of product 2. (d) Crystal structures of 2 (CCDC 2513352). Ellipsoids are shown at 50% probability level.

Although the products are known, full spectral analysis is not reported in the literature for 4. In addition, no direct conversion of β-naphthol to this product (4) is reported in the literature. One single report dealing with the synthesis of 4 goes back to 1951, where Bader reported that 40 h treatment of 1 in H_2_O_2_/glacial AcOH solution produced 53% of 4.^43^ However, in addition to the melting point, no spectral characterization was given for the product at the time. This background urged us to conduct the full analysis of 4. In the ^1^H NMR spectrum, the presence of a broad peak at 12.9 ppm with the integration of one proton was the indication for the presence of a carboxylic acid group in the product. The remaining eleven aromatic peaks were also distributed from low to high field as six doublet (d), four doublet of doublet (dd), and a singlet (s) protons (Fig. 5a). This would correspond to the proposed structure and was further verified with ^13^C NMR (full and the DEPT-90, Fig. 5b) and the FTIR spectroscopies (Fig. 5c). In addition, we obtained the single crystal of 4 and subjected it to X-ray analysis (Fig. 5d) to vigorously confirm the formation of the tetracyclic product 4.

It should be noted that the oxidative performance of H_2_O_2_ is highly dependent on the reaction conditions and the nature of the catalyst,^44^ allowing H_2_O_2_ to be efficiently activated under different conditions. In other words, activation of H_2_O_2_ with acids, bases, or metal oxides may lead to the generation of highly reactive species such as peracids, hydroperoxide ion, or metal-peroxo intermediates.^45^

**Fig. 6:**
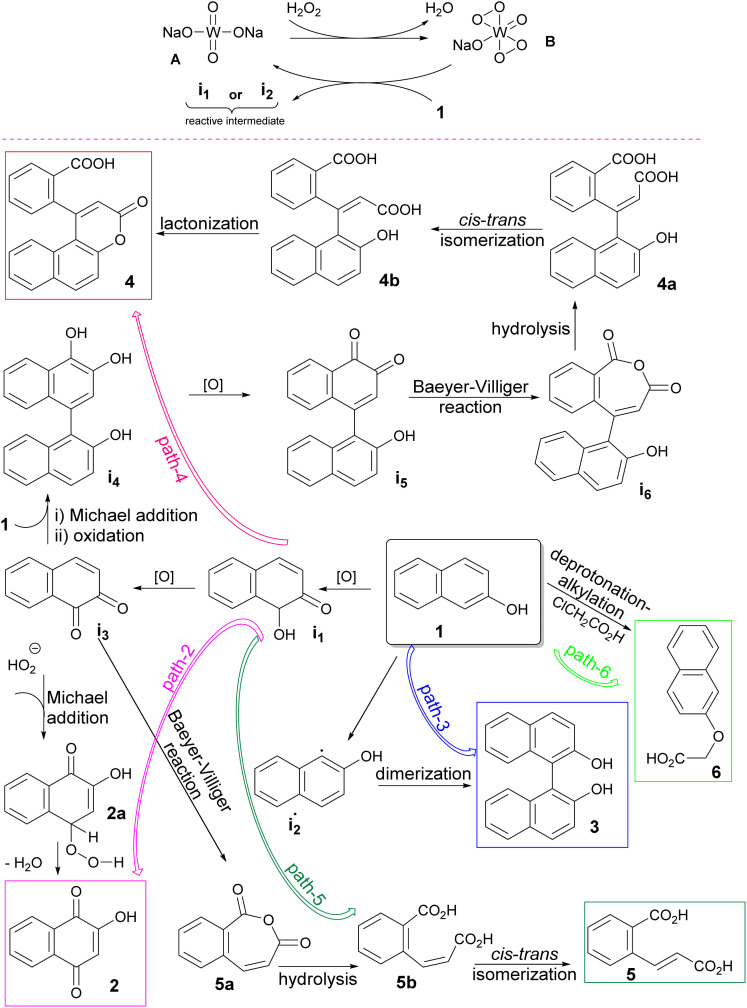
The proposed mechanism of the reactions.

Based on these, a simplified mechanism can be proposed for the transformations of 1 to 2–6 (Fig. 6). Initially, sodium tungstate A is converted by H_2_O_2_ into peroxytungstate species B, these species could in turn convert β-naphthol into reactive intermediates i_1_ and i_2_, depending on the use of either acidic or basic conditions, respectively. Under alkaline conditions, hydrogen peroxide would provide HO_2_^−^ species that can undergo Michael addition to intermediate i_3_ (path-2) to result in Lawsone 2.^46^ In contrast, by the use of a mild dilute acetic acid (p*K*_a_ = 4.76) solution, a radical pathway would dominate for selective oxidative C–C dimerization of 1, leading to the formation of BINOL 3 (path-3).^47^ By increasing the concentration of acetic acid, a sequential oxidative Michael addition (i_3_ → i_4_)-Baeyer–Villiger ring expansion (i_5_ → i_6_)-hydrolysis (i_6_ → 4a)-isomerization (4a → 4b)-lactonization to 4 would occur (path-4).^48^ When the system is switched to the stronger formic acid (p*K*_a_ = 3.75) condition, the initially formed i_3_ would directly undergo oxidative Baeyer–Villiger ring expansion to convert to 5, after a hydrolysis-isomerization sequence of transformations (path-5).^49^ With 2-chloroacetic acid, direct nucleophilic replacement of chlorine with the naphtholate anion would dominate, due to the high electrophilicity of ClCH_2_CO_2_H (path-6).^50^ Appropriate citations were provided to support the proposed mechanism, available in the literature for similar transformations.

## Experimental

### General

FTIR spectra were recorded using KBr disks on a Bruker Vector-22 spectrometer. NMR spectra were obtained on a Qone (400 MHz for ^1^H and 100 MHz for ^13^C) as CDCl_3_ solutions using TMS as internal standard reference. Elemental analyses were performed using a Thermo Finnigan Flash EA 1112 instrument. MS spectra were obtained on a Shimadzu LCMS 2010 A using electrospray ionization. TLC experiments were carried out on pre-coated silica gel plates using petroleum ether/EtOAc as the eluent. Starting materials and reagents were purchased from commercial sources.

### NaOH-catalyzed, Na_2_WO_4_·2H_2_O-promoted oxidation of 1 to 2

A mixture of β-naphthol (300 mg, 2 mmol), NaOH (2.0 mL, 40% w/v), and Na_2_WO_4_·2H_2_O (10 mg, 0.03 mmol, 1.5 mol%) was stirred at rt for 10–15 min until a clear solution was obtained. To this mixture was added H_2_O_2_ (1.0 mL, 30% w/v) dropwise, while the temperature of the mixture was kept below 40 °C. The mixing was continued until the temperature of the reaction mixture reached to rt. At this stage, TLC monitoring of the reaction (using silica gel coated plates and a 4 : 1 EtOAc/hexanes mixture as the eluent) showed complete disappearance of the starting reactant. Then, HCl_aq_ solution (5% w/v) was added to the mixture to adjust the pH to about 7.0. The orange solid product (mp = 192–193 °C; 320 mg, 92%) was isolated by filtration and recrystallized from hot EtOH. The filtered catalyst was recovered and reused in the next reactions. FTIR (KBr) 3170, 1640, 1582 cm^−1^; ^1^H NMR (400 MHz, DMSO-*d*_6_), *δ* = 6.15 (s, 1H), 7.77 (ddd, *J* = 1.0, 6.0, 7.0 Hz, 1H), 7.81 (ddd, *J* = 1.0, 6.0, 7.0 Hz, 1H), 7.91 (dd, *J* = 1.0, 6.0 Hz, 1H), 7.97 (dd, *J* = 1.0, 6.0 Hz, 1H), 11.65 (br s, 1H) ppm; ^13^C NMR (100 MHz, DMSO-*d*_6_) *δ* = 111.5, 125.9, 126.4, 131.1, 132.4, 133.7, 134.9, 160.0, 181.7, 185.1.

### AcOH (dil.)-Na_2_WO_4_·2H_2_O-promoted oxidation of 1 to 3

A mixture of 1 (300 mg, 2 mmol), AcOH (2.0 mL, 10% w/v), and Na_2_WO_4_·2H_2_O (10 mg, 0.03 mmol, 1.5 mol%) was stirred at rt for 10–15 min until a clear solution was obtained. To this mixture was added H_2_O_2_ (1.0 mL, 30% w/v) dropwise, while the temperature of the mixture was kept below 40 °C. The mixing was continued until the temperature of the reaction mixture reached to rt. At this stage, TLC monitoring of the reaction confirmed the complete disappearance of the starting reactant. Then, a saturated solution of NaHCO_3_ was added to the mixture to adjust the pH at about 7.0. The cream solid product (mp = 212–214 °C; 266 mg, 93%) was obtained by filtration and recrystallization from hot EtOH. The filtered catalyst was recovered and reused in the next reactions. FTIR (KBr) 3482, 3400, 3046, 1586 cm^−1^; ^1^H NMR (400 MHz, CDCl_3_), *δ* = 5.07 (br s, 2H), 7.19 (d, *J* = 8.0 Hz, 2H), 7.28–7.36 (m, 2H), 7.39–7.43 (m, 4H), 7.92 (d, *J* = 8.0 Hz, 2H), 8.00 (d, *J* = 9.0 Hz, 2H) ppm; ^13^C NMR (100 MHz, CDCl_3_) *δ* = 110.9, 117.8, 124.1, 124.2, 127.5, 128.4, 129.5, 131.5, 133.4, 152.8.

### AcOH (conc.)-Na_2_WO_4_·2H_2_O-promoted oxidation of 1 to 4

A mixture of 1 (300 mg, 2 mmol), AcOH (2.0 mL, 40% w/v), and Na_2_WO_4_·2H_2_O (10 mg, 0.03 mmol, 1.5 mol%) was stirred at rt for 10–15 min to obtain a clear solution. To this mixture was added H_2_O_2_ (1.0 mL, 30% w/v) dropwise, while the temperature of the mixture was kept below 40 °C. The mixing was continued until the temperature of the reaction mixture reached to rt. At this stage, TLC monitoring of the reaction showed complete disappearance of the reactant. Then, a saturated NaHCO_3_ solution was added to the mixture to adjust the pH at about 7.0. The white solid product (mp = 273–275 °C; 300 mg, 95%) was obtained by filtration and recrystallization from hot EtOH. The filtered catalyst was recovered and reused in the next reactions.

2-(3-Oxo-3*H*-benzo[*f*]chromen-1-yl)benzoic acid (4). White crystals, 95% (300 mg); 273–275 °C; FTIR (KBr) 3052, 1690, 1675, 1079 cm^−1^; ^1^H NMR (400 MHz, DMSO-*d*_6_), *δ* = 6.29 (s, 1H), 7.02 (d, *J* = 9.0 Hz, 1H), 7.19 (dd, *J* = 7.5, 7.5 Hz, 1H), 7.44 (dd, *J* = 7.5, 7.5 Hz, 1H), 7.46 (d, *J* = 7.5 Hz, 1H), 7.67 (d, *J* = 9.0 Hz, 1H), 7.76 (dd, *J* = 8.0, 8.0 Hz, 1H), 7.83 (dd, *J* = 8.0, 8.0 Hz, 1H), 8.02 (d, *J* = 8.0 Hz, 1H), 8.16 (d, *J* = 7.5 Hz, 1H), 8.23 (d, *J* = 9.0 Hz, 1H), 12.92 (br s, 1H) ppm; ^13^C NMR (100 MHz, DMSO-*d*_6_) *δ* = 113.9, 115.0, 117.9, 124.4, 125.8, 127.7, 129.4, 129.5, 129.8, 129.9, 131.3, 131.4, 133.6, 134.3, 140.8, 154.2, 157.9, 159.7, 167.2 ppm; ESI-MS: *m*/*z* 315 [M–H]^−^; anal. calcd for C_20_H_12_O_4_: C, 75.94; H, 3.82. Found: C, 75.77; H, 3.92.

### HCO_2_H–Na_2_WO_4_·2H_2_O-promoted oxidation of 1 to 5

A mixture of 1 (300 mg, 2 mmol), HCO_2_H (2 mL, 10%), and Na_2_WO_4_·2H_2_O (10 mg, 0.03 mmol, 1.5 mol%) was stirred at rt for 10–15 min to obtain a clear solution. To this mixture was added H_2_O_2_ (1.0 mL, 30% w/v) dropwise, while the temperature of the mixture was kept below 5 °C. The mixing was continued until the temperature of the reaction mixture reached to rt. The progress of the reaction was monitored by TLC using EtOAc/hexanes (4 : 1) as the eluent. Then, 2 mL of a saturated NaHCO_3_ solution was added to neutralize the reaction mixture and dissolve the product. The catalyst was removed from the mixture by simple filtration and the filtrate was acidified by HCOOH to get back the product as a solid. The precipitate was collected by filtration, washed with water, dried, and recrystallized from hot EtOH to afford the white product (mp = 170–171 °C; 361 mg, 94%). FTIR (KBr) 2980, 1697, 1272 cm^−1^; ^1^H NMR (400 MHz, DMSO-*d*_6_), *δ* = 6.43 (d, *J* = 16.0 Hz, 1H), 7.52 (ddd, *J* = 1.0, 7.5, 7.5 Hz, 1H), 7.62 (ddd, *J* = 1.5, 7.5, 7.5 Hz, 1H), 7.83 (dd, *J* = 1.0, 7.5, 7.5 Hz, 1H), 7.89 (d, *J* = 1.5, 7.5 Hz, 1H), 8.32 (d, *J* = 16.0 Hz, 1H), 12.83 (br s, 2H) ppm. ^13^C NMR (101 MHz, DMSO-*d*_6_) *δ* 121.9, 128.2, 130.2, 130.8, 131.6, 132.5, 135.3, 143.0, 167.9, 168.6.

### Synthesis of 2-(naphthalen-2-yloxy)acetic acid 6

A mixture of 1 (300 mg, 2.0 mmol), 2-ClCH_2_CO_2_H (189 mg, 2.0 mmol), and Na_2_WO_4_·2H_2_O (10 mg, 0.03 mmol, 1.5 mol%) in H_2_O (3.0 mL) was stirred at rt for 20 min to obtain a clear solution. TLC monitoring of the reaction confirmed the complete disappearance of the starting reactant. Then, NaHCO_3sat_ solution was added to the mixture to adjust the pH to the neutral region. The cream solid product (mp = 150 °C decomp.; 364 mg, 92%) was obtained by filtration and recrystallization from hot EtOH. The filtered catalyst was recovered and reused in the next reactions. FTIR (KBr) 2906, 1737, 1252 cm^−1^; ^1^H NMR (400 MHz, DMSO-*d*_6_), *δ* = 13.03 (br s, 1H), 7.87–7.85 (m, 2H), 7.81 (d, *J* = 8.0 Hz, 1H), 7.48 (ddd, *J* = 1.5, 7.5, 7.5 Hz, 1H), 7.37 (ddd, *J* = 1.1, 7.5, 7.5 Hz, 1H), 7.29–7.27 (m, 1H), 7.22 (d, *J* = 2.5, 9.0 Hz, 1H), 7.22 (dd, *J* = 2.5, 9.0 Hz, 1H), 4.82 (s, 2H) ppm; ^13^C NMR (100 MHz, DMSO-*d*_6_) *δ* 65.1, 107.5, 118.9, 124.3, 126.9, 127.2, 128.0, 129.2, 129.9, 134.6, 156.1, 170.6.

### X-ray crystallography

Crystals of 4 suitable for single-crystal X-ray diffraction analysis were obtained by slow evaporation, dissolving 120 mg of the compound in 10 mL of ethanol. The X-ray diffraction measurements were made on a STOE IPDS-2T diffractometer with graphite monochromated Mo-Kα radiation. For complex 4, colorless plate shape single-crystal was chosen using a polarizing microscope and was mounted on a glass fiber which was used for data collection. Cell constants and orientation matrices for data collection were obtained by least-squares refinement of diffraction data from 3880 unique reflections. Data were collected to a maximum 2*θ* value of 58.44° in a series of *ω* scans in 1° oscillations and integrated using the Stoe X-AREA^51^ software package. The data were corrected for Lorentz and polarizing effects. The structures were solved by direct methods^52^ and subsequent difference Fourier maps and then refined on *F*^2^ by a full-matrix least-squares procedure using anisotropic displacement parameters.^53^ The atomic factors were taken from the international tables for X-ray crystallography.^54^ All refinements were performed using the X-STEP32 crystallographic software package.^55^ Relevant crystallographic data are reported in Table 2.

**Table 2:** Crystal data and structure refinement for 4

Compound	4
Empirical formula	C_20_H_12_O_4_
Formula weight	316.30
Temperature	298(2) K
Wavelength	0.71073 Å
Crystal system, space group	Monoclinic, *P*2_1_/*c*
Unit cell dimensions	*a* = 11.969(2) Å alpha = 90 deg.
	*b* = 7.8791(16) Å beta = 102.95(3) deg.
	*c* = 15.786(3) Å gamma = 90 deg.
Volume	1450.9(5) Å^3^
Z, calculated density	4, 1.448 Mg m^−3^
Absorption coefficient	0.101 mm^−1^
*F*(000)	656
Crystal size	0.5 × 0.2 × 0.15 mm
Theta range for data collection	1.75 to 29.22 deg.
Limiting indices	−16 ≤ *h* ≤ 16, −10 ≤ *k* ≤ 10, −21 ≤ *l* ≤ 21
Reflections collected/unique	10 734/3880 [*R*(int) = 0.1131]
Completeness to theta = 29.22	98.3%
Absorption correction	None
Refinement method	Full-matrix least-squares on *F*^2^
Data/restraints/parameters	3880/1/221
Goodness-of-fit on *F*^2^	1.019
Final *R* indices [*I* > 2sigma(*I*)]	*R*1 = 0.0569, w*R*2 = 0.1375
R indices (all data)	*R*1 = 0.0849, w*R*2 = 0.1504
Largest diff. peak and hole	0.267 and −0.229 e. Å^−3^
CCDC no.	2513352

## Conclusion

**Fig. 7:**
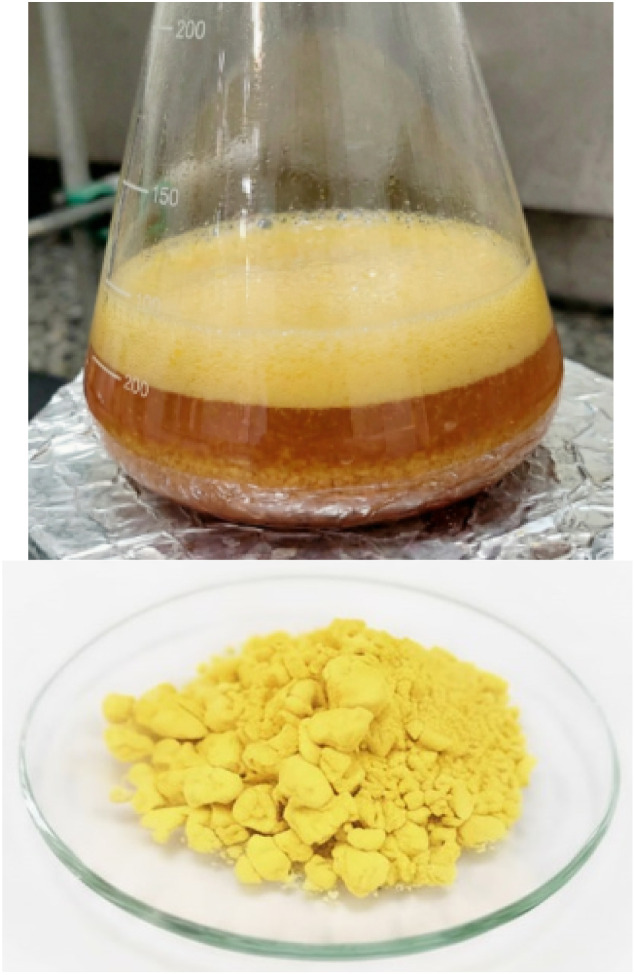
Large-scale (10 grams) preparation of Lawsone: neutralization using HCl (top) and isolation (94%) of the pure product (bottom).

The aqueous-phase oxidation of β-naphthol using H_2_O_2_ in the presence of sodium tungstate demonstrated a highly tunable, condition-dependent process leading to the selective formation of products of choice. Thus, careful control of the reaction conditions and the pH of the medium allowed precise access to structurally diverse products (2–6) in high yields. Under basic conditions, Lawsone 2 formed rapidly and efficiently, whereas mild acidic conditions promoted selective dimerization of 1 to 1,1′-bi-2-naphthol 3. Higher acid concentrations facilitated an oxidation–dimerization–cyclization cascade sequence to produce benzochromene 4. At the same time, the use of formic acid or chloroacetic acid shifted the selectivity toward the formation of products 5 and 6, respectively. These results highlighted the pivotal role of aqueous medium and pH control in dictating reaction pathways, offering operational simplicity, short reaction times, and scalability. Furthermore, the catalyst exhibited excellent recyclability and maintained high efficiency over multiple cycles, underscoring the practical applicability of this methodology for the selective synthesis of diverse β-naphthol-related products. Comparative evaluation of the results of the present work with some other related recent studies for the synthesis of each of the products is summarized in Table 3. The comparison clearly demonstrates the efficiency of the current method in terms of operational simplicity, short reaction times, and high product yields, when compared to previously reported multi-step or time-consuming procedures. The aqueous reaction medium not only facilitates environmentally benign conditions but also allows precise control over product selectivity through simple adjustments of pH and reaction parameters. Collectively, the method presents a new approach for selective high-scale synthesis of β-naphthol-derived compounds. Furthermore, the feasibility of the method to prepare high-scale products is exemplified in Fig. 7 for high yield synthesis of Lawsone on a 10 gram scale.

**Table 3:** Comparative evaluation of the developed method and previously reported methods

Entry	Reagents, temperature, time	Solvent	Product	Yield%	References
1	H_2_O_2_, NaOH, rt, 45 min	H_2_O	2	92	This work
2	(i) Me_2_SO_4_, K_2_CO_3_, dry acetone, 56 °C, 1 h; (ii) DMF-POCl_3_, 0 °C-rt-120 °C, 4 h; (iii) NBS, DMF-H_2_O (95 : 5), rt, 16 h; (iv) AlCl_3_, CH_2_Cl_2_, 40 °C, 3 h	—	2	57	Kumar *et al.*^56^
3	NaOH, O_2_, tetra(4-methoxyl-phenyl)porphyrinate iron(iii) chloride, 50 °C, 9 h	MeOH	2	33	Yan *et al.*^57^
4	H_2_O_2_, NaOH, 5,10,15,20-tetrakis(*p*-sulfonatophenyl) porphinatomanganese(iii) chloride, 25 °C, 15 min	H_2_O	2	95	Hassanein *et al.*^46^
5	H_2_O_2_, CH_3_CO_2_H, rt, 15 min	H_2_O	3	93	This work
6	CuI (10 mol%), *N*-isopropyl-1-(pyridin-2-ylmethyl)piperidine-2-carboxamide (20 mol%), air	MeCN	3	77	Tian *et al.*^58^
7	H_2_O_2_, Pd–Au–zeolite-Y, 60 °C, 4 h	PhCH_3_	3	58	Sharma *et al.*^59^
8	Air, KOH, 10 °C, 3 h; rt, 4 days	MeOH	3	62	Jafroodi *et al.*^60^
9	H_2_O_2_, CH_3_CO_2_H, rt, 40 min	H_2_O	4	95	This work
10	(i) H_2_O_2_, (NH_4_)_6_Mo_7_O_24_, glacial acetic acid, EtOH, 3 h (ii) H_2_O_2_, glacial acetic acid, rt, 40 h	EtOH	4	39	Bader *et al.*^43^
11	H_2_O_2_, Na_2_MoO_4_, glacial acetic acid, 0 °C, 25 h		4	5	Raacke-Fels *et al.*^61^
12	H_2_O_2_, HCO_2_H, rt, 30 min	H_2_O	5	94	This work
13	H_2_O_2_ (10.0 eq.), Na_2_WO_4_·2H_2_O (5 mol%), 10 °C, 102 h	MeCN	5	55	Liu *et al.*^62^
14	Oxone (2KHSO_5_·KHSO_4_·K_2_SO_4_), rt, 10 h	MeCN, H_2_O	5	80	Parida *et al.*^63^
15	H_2_O_2_, (PhSe)_2_, 55 °C, 6 h	*t*-BuOH	5	60	Giurg *et al.*^49^
16	NaOH, CH_2_ClCO_2_H, rt, 45 min	H_2_O	6	90	This work
17	NaOH, CH_2_ClCO_2_H, 70 °C, 15 h	H_2_O	6	83	Kumari *et al.*^64^
18	NaOH, MW-US reactor, 100 °C, 10 min	H_2_O	6	91	Pawełczyk *et al.*^65^
19	NaOH, 60 °C, 7 h	H_2_O	6	90	del Carmen Cruz *et al.*^50^

## Author contributions

M. M. Mojtahedi conceived and designed the work. E. Ashoori performed the experiments and collected data. M. S. Abaee reviewed the draft and performed the literature survey. All authors analyzed the data, discussed the results, and reviewed the manuscript.

## Conflicts of interest

There are no conflicts to declare.

## Supplementary Material

RA-016-D6RA03782H-s001

RA-016-D6RA03782H-s002

## Data Availability

CCDC 2513352 (2) contains the supplementary crystallographic data for this paper.^66^ Copies of the spectra of products are available in the supplementary information (SI) of this article. Supplementary information is available. See DOI: https://doi.org/10.1039/d6ra03782h.
