# Supplementary material for: Aqueous Na2WO4/H2O2: an efficient tunable catalytic medium for selective oxidation of β-naphthol to diverse products
Source: RSC Adv. 2026 Jul 6;16(36):38054–64. doi: 10.1039/d6ra03782h (PMC13335222; doi:10.1039/d6ra03782h)
Supplement: Supplementary file 1 [file RA-016-D6RA03782H-s001.pdf]

Supplementary Information (SI) for RSC Advances.
This journal is © The Royal Society of Chemistry 2026

Aqueous Na_2WO_4/H_2O_2: an efficient tunable catalytic medium for selective oxidation of β-naphthol to diverse products

Mohammad M. Mojtahedi, Elham Ashoori, M. Saeed Abaee

Department of Organic Chemistry and Natural Products, Chemistry and Chemical Engineering Research Center of Iran, Pajouhesh Blvd, 17th Km Tehran-Karaj Highway, P.O.Box 14335-186, Tehran, Iran

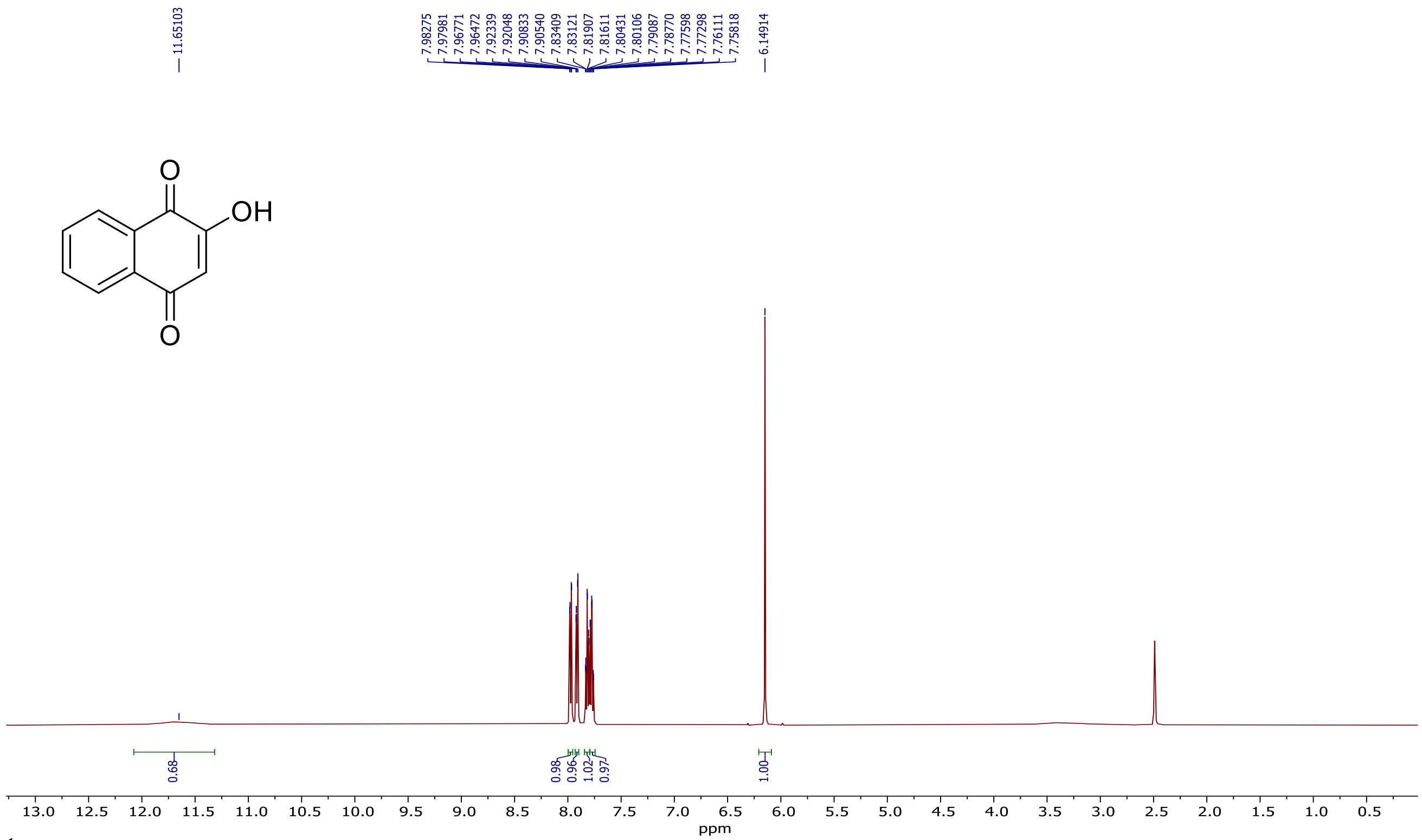

^{1}H NMR (400 MHz, DMSO-d_6); Lawsone **2**.

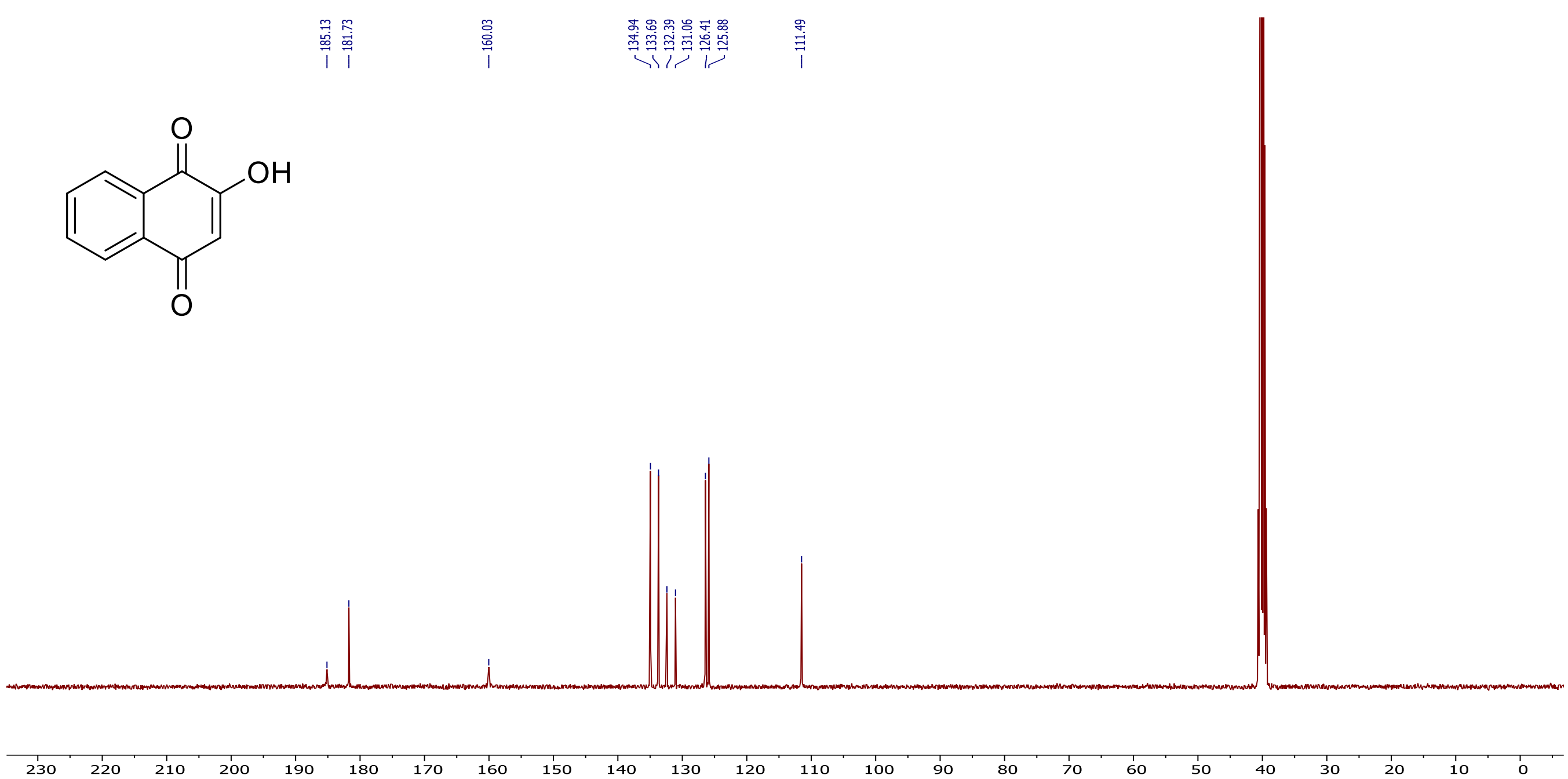

^{13}C NMR (100 MHz, DMSO-d_6); Lawsone **2**.

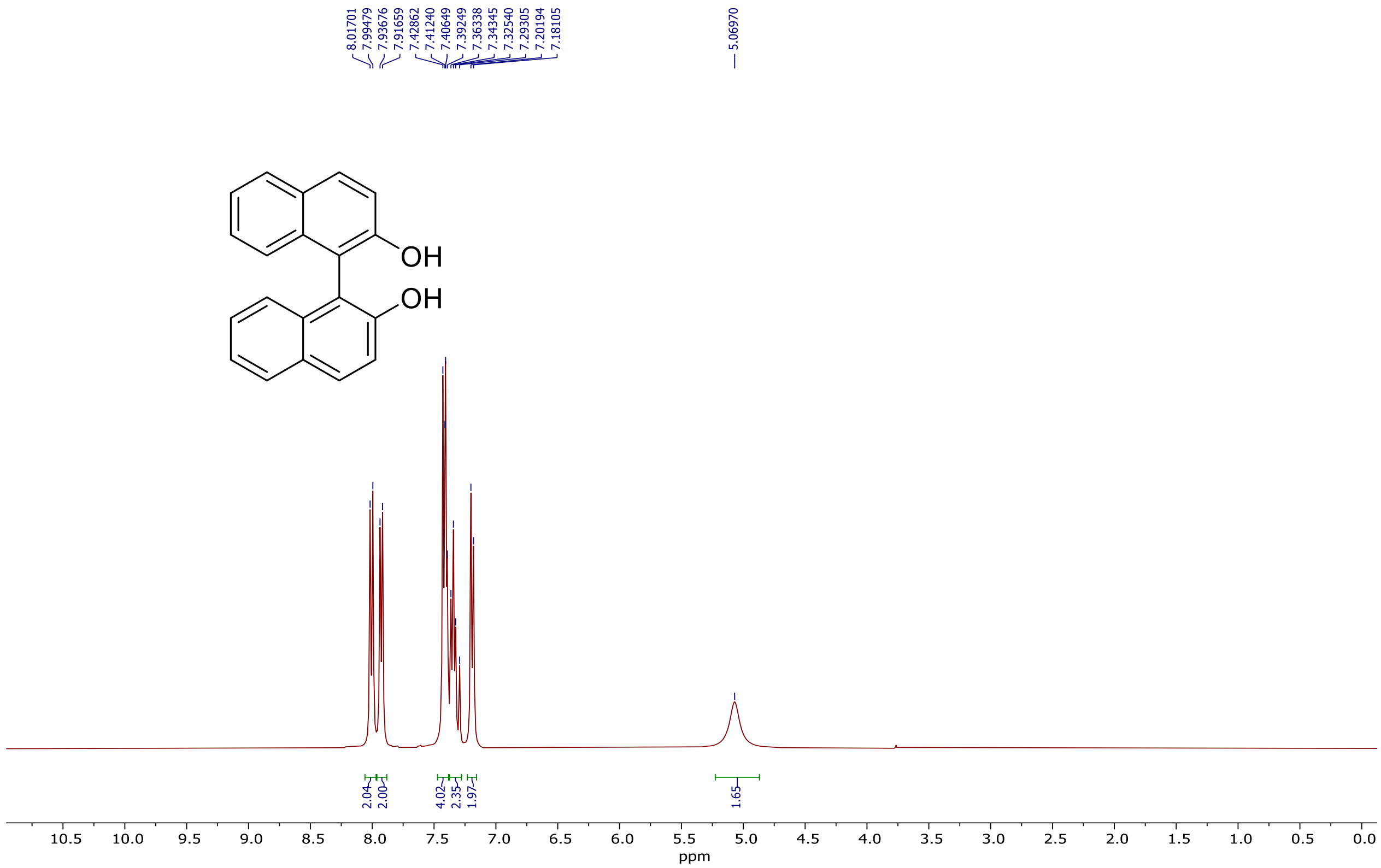

¹H NMR (400 MHz, $CDCl_3$); [1,1'-Binaphthalene]-2,2'-diol **3**.

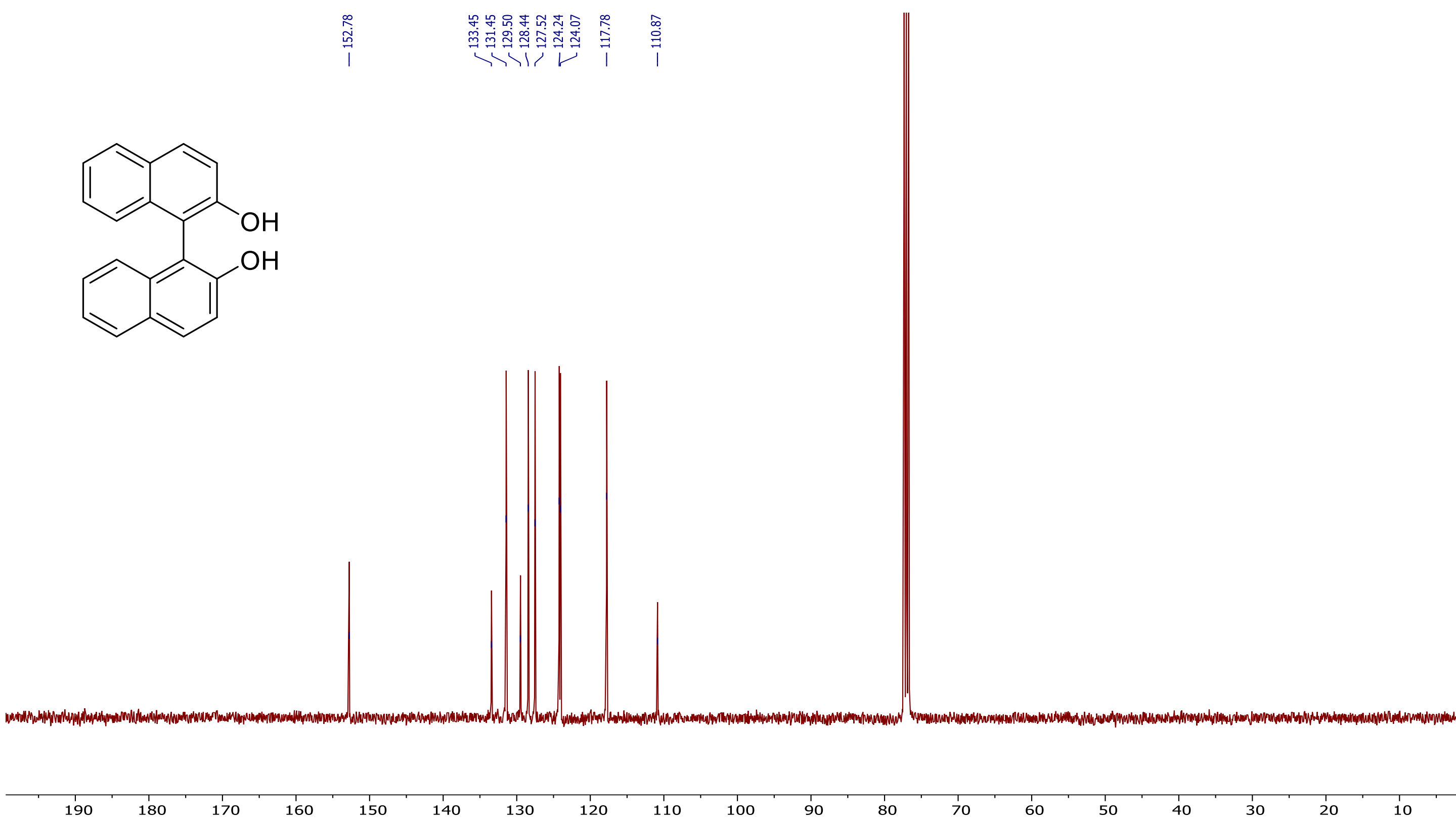

¹³C NMR (100 MHz, $CDCl_3$); [1,1'-Binaphthalene]-2,2'-diol **3**.

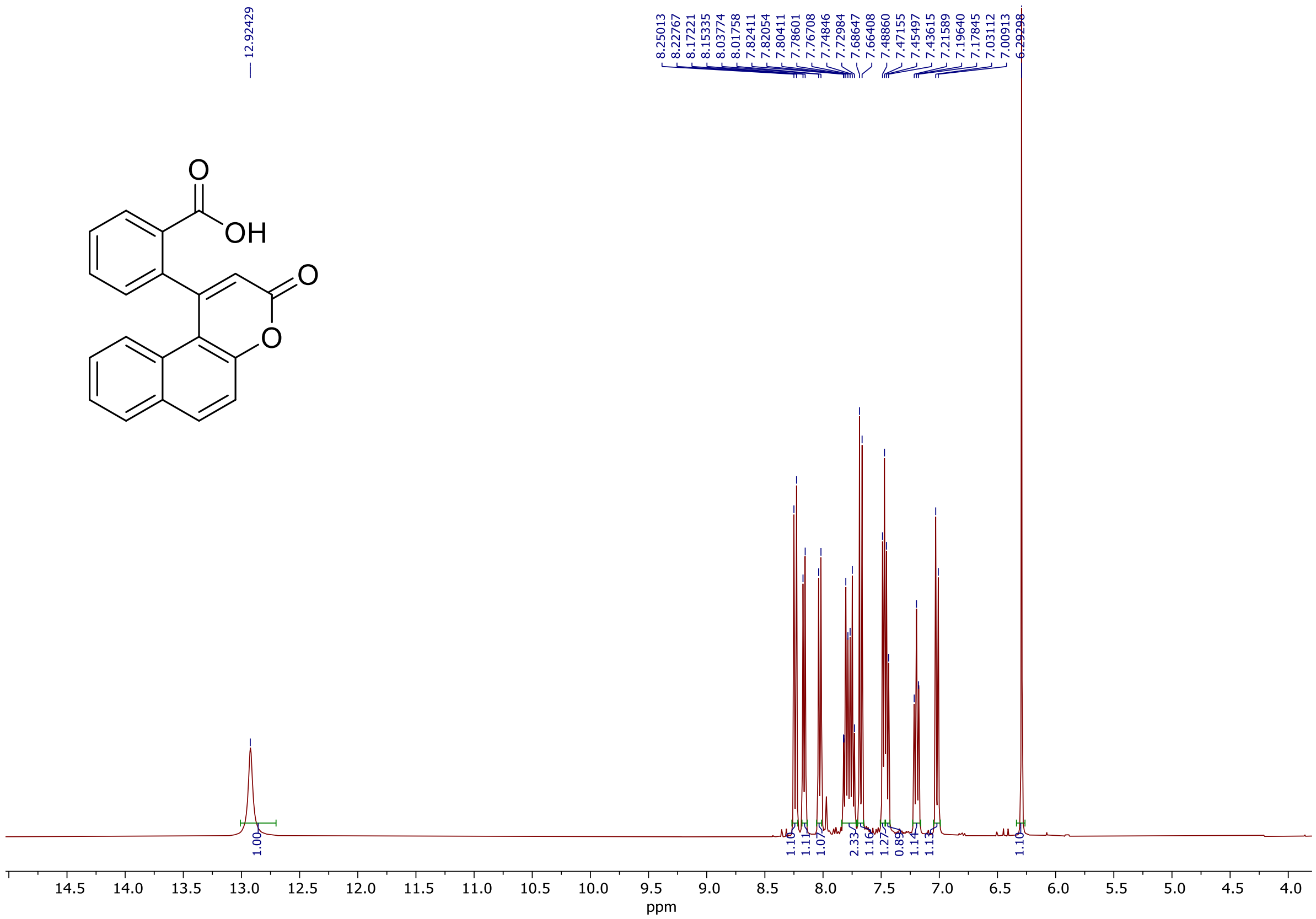

^{1}H NMR (400 MHz, DMSO-d_6); 2-(3-Oxo-3*H*-benzo[*f*]chromen-1-yl)benzoic acid **4**.

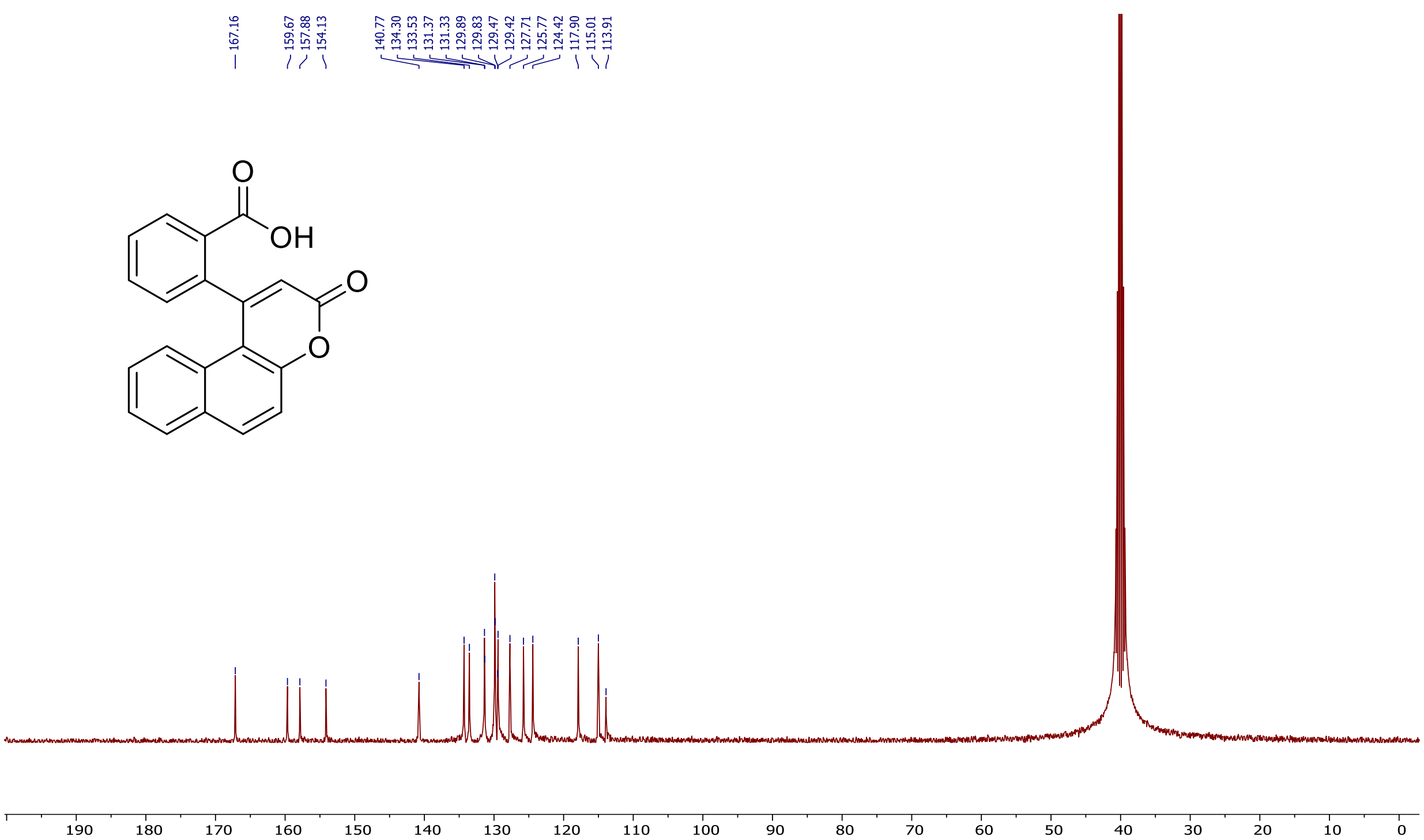

^{13}C NMR (100 MHz, DMSO-d_6); 2-(3-Oxo-3*H*-benzo[*f*]chromen-1-yl)benzoic acid **4**.

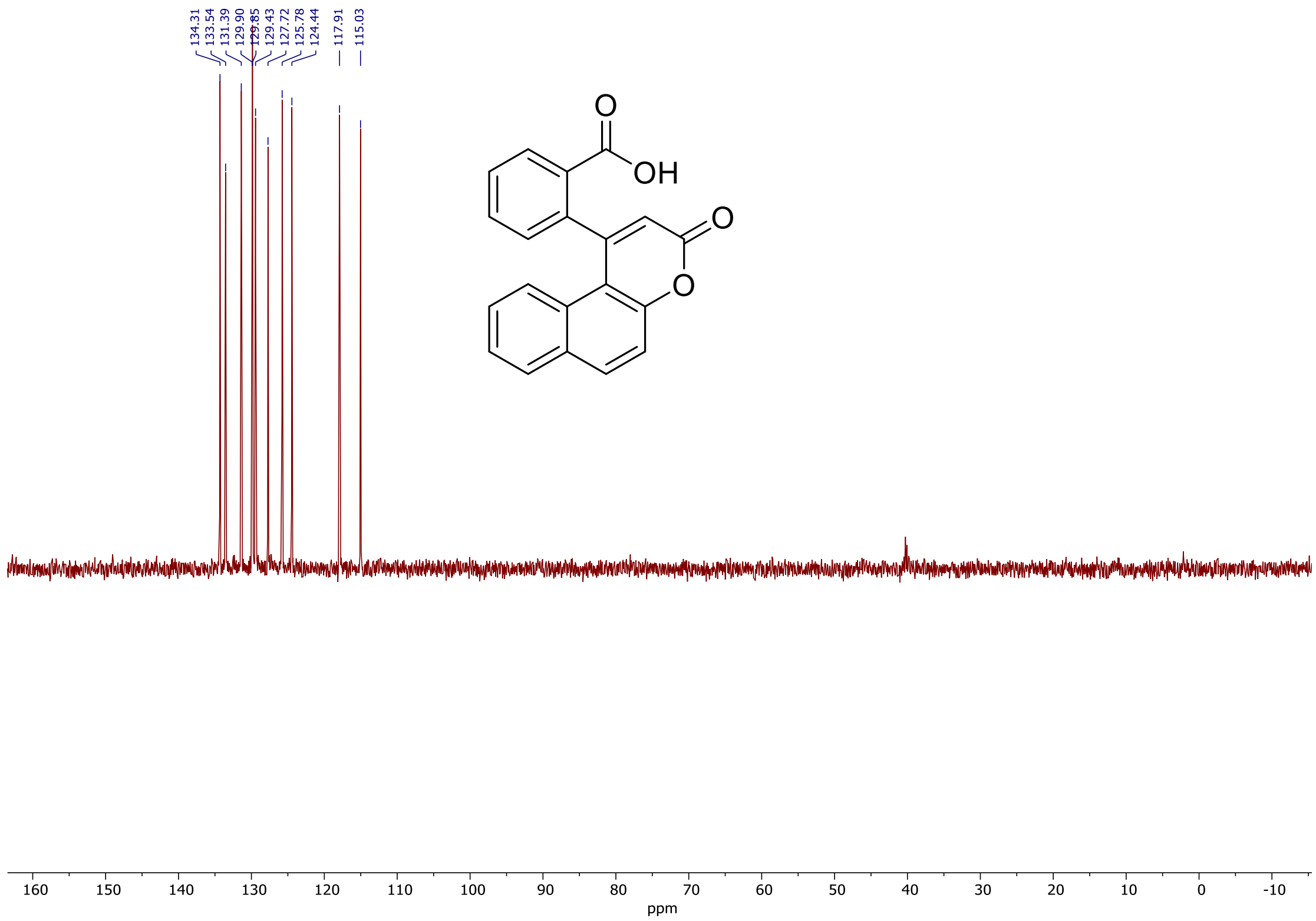

^{13}C DEPT 135 NMR (100 MHz, DMSO-d_6); 2-(3-Oxo-3*H*-benzo[*f*]chromen-1-yl)benzoic acid **4**.

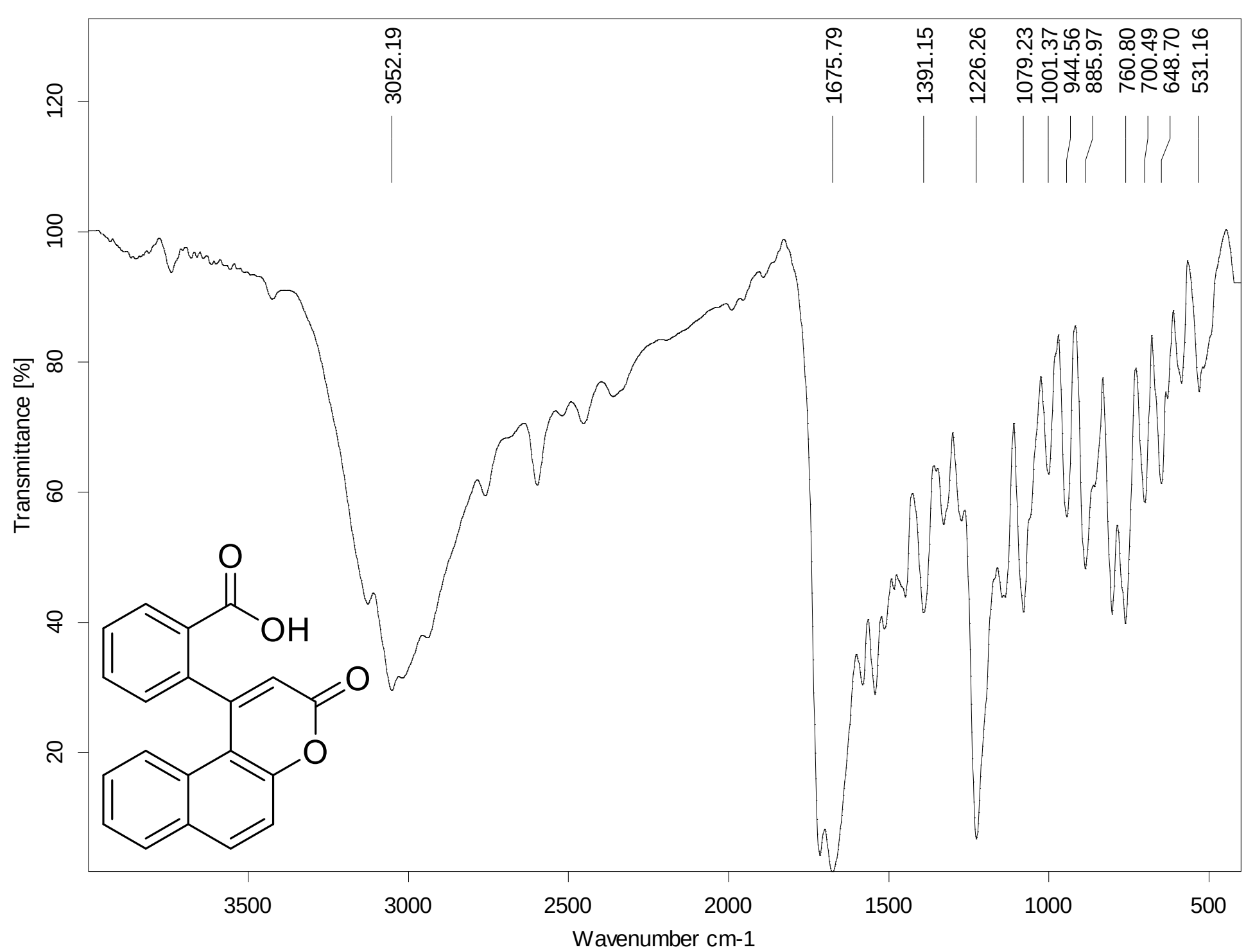

FTIR (KBr, cm^{-1}); 2-(3-Oxo-3*H*-benzo[*f*]chromen-1-yl)benzoic acid **4**.

ESI–MS (negative mode); 2-(3-Oxo-3*H*-benzo[*f*]chromen-1-yl)benzoic acid **4**.

Analysis conditions

LC conditions:

- Mobile phase:

**Solvent A**	**ACN + 0.1 % Formic Acid**	**50%**
**Solvent B**	**H_2O+ 0.1 % Formic Acid**	**50%**

- Flow rate: 0.3 ml/min
- Injection volume: 20 µL

MASS conditions:

- Mode: ESI+
- Detection gain: 1.8 kV
- Prob Volt: 4.5 kV
- CDL Volt: 30 V
- Gas nebulizer: N_2 (grade 5)
- Flow gas: 1.2 L/min
- CDL temperature: 240 ºC
- Block temperature: 270 ºC

Device model:

Shimadzu LCMS 2010 A

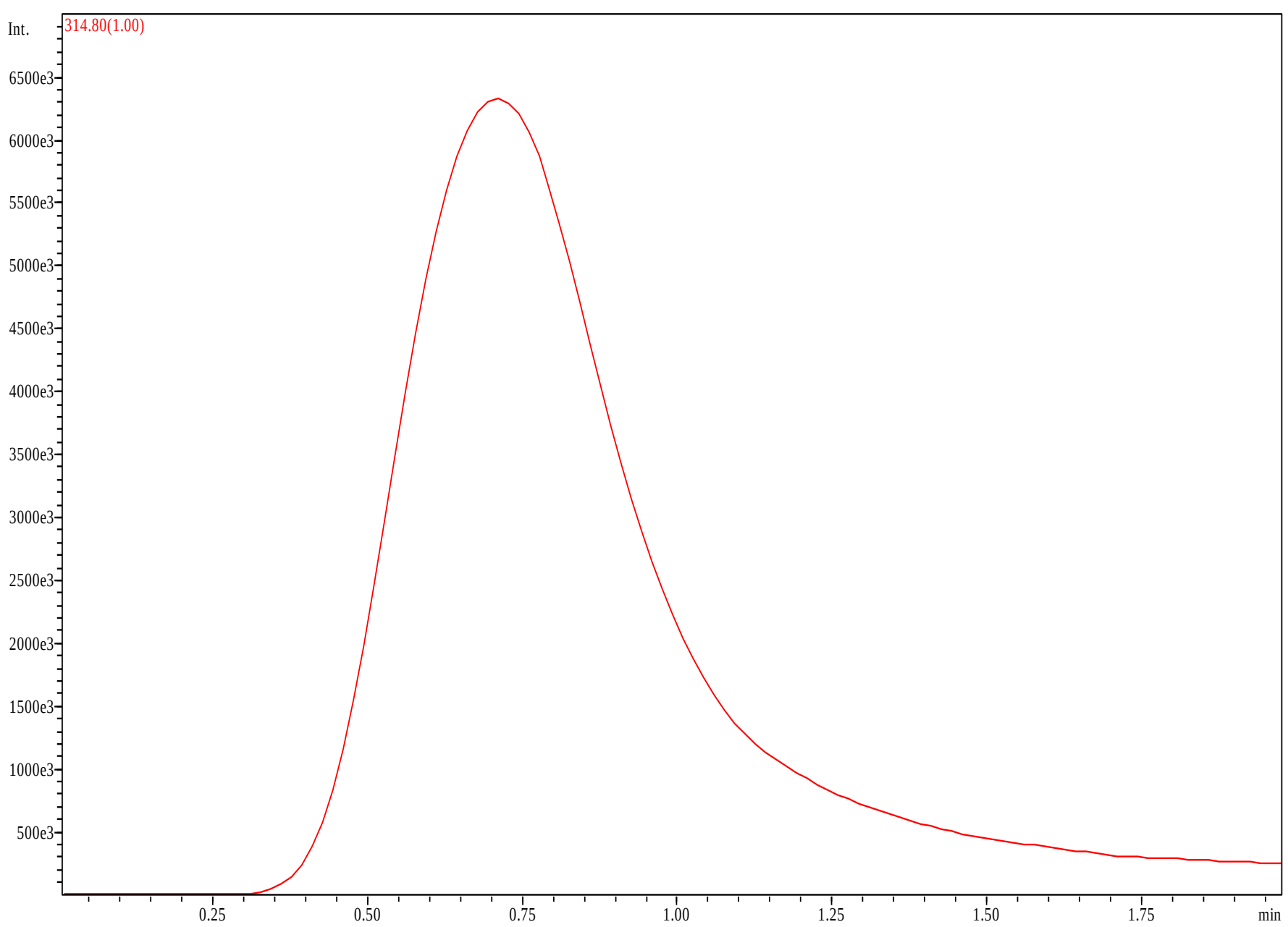

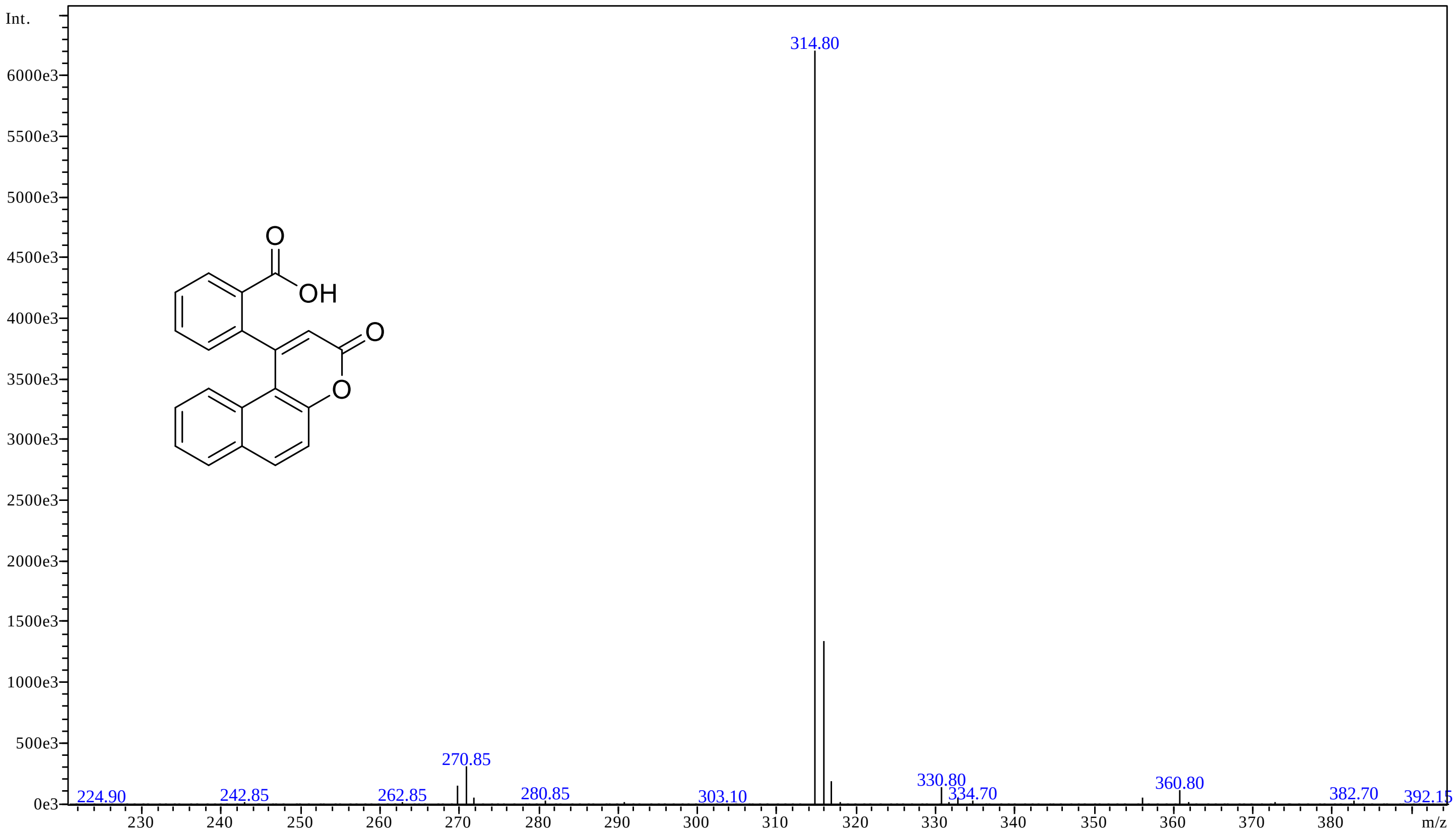
Int.
6000e3
5500e3
5000e3
4500e3
4000e3
3500e3
3000e3
2500e3
2000e3
1500e3
1000e3
500e3
0e3
314.80
270.85
330.80
334.70
360.80
224.90
242.85
262.85
280.85
303.10
382.70
392.15
230
240
250
260
270
280
290
300
310
320
330
340
350
360
370
380
m/z
O
OH
O
O

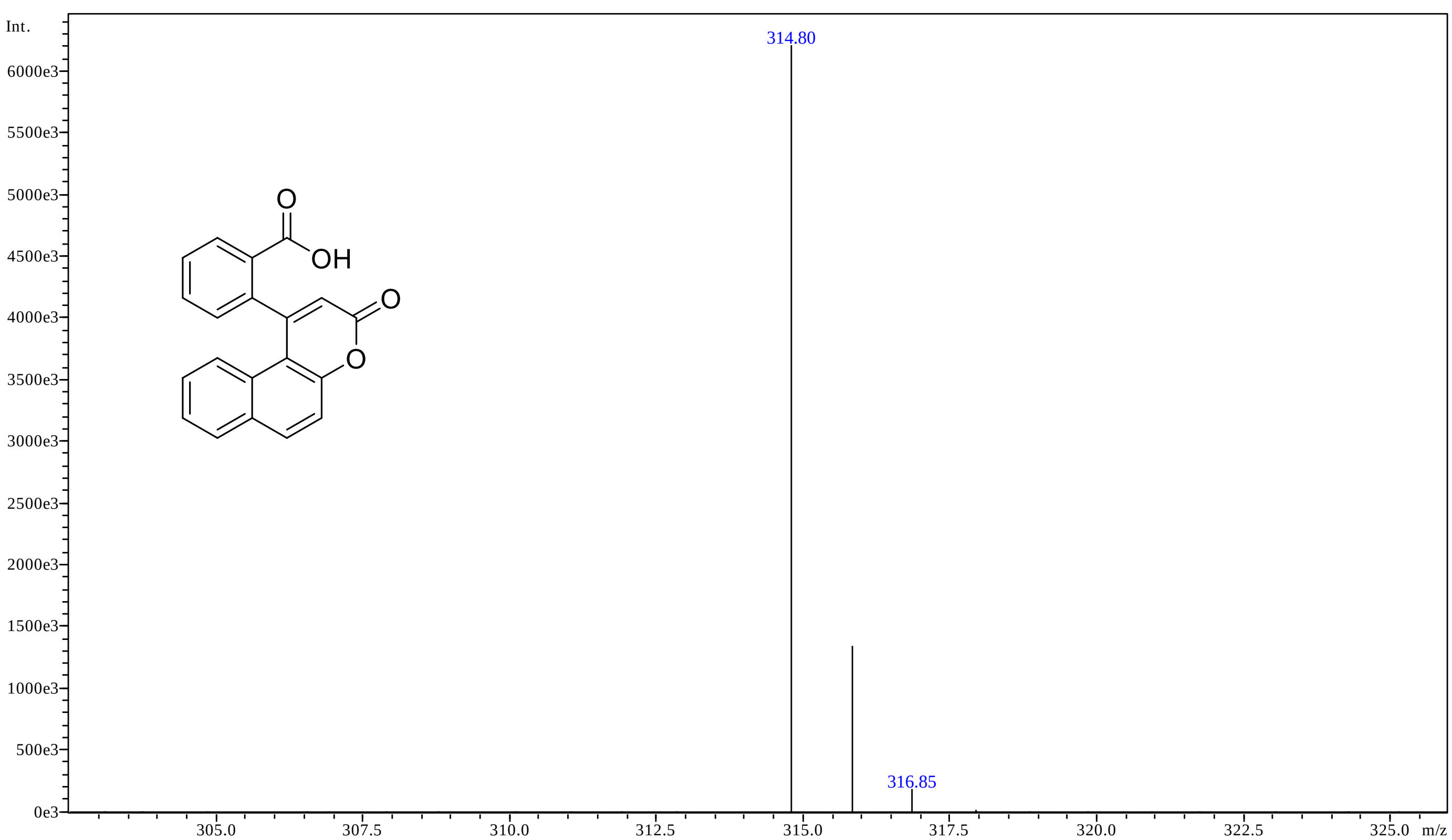
Int.
6000e3
5500e3
5000e3
4500e3
4000e3
3500e3
3000e3
2500e3
2000e3
1500e3
1000e3
500e3
0e3
314.80
316.85
305.0
307.5
310.0
312.5
315.0
317.5
320.0
322.5
325.0
m/z
O
OH
O
O

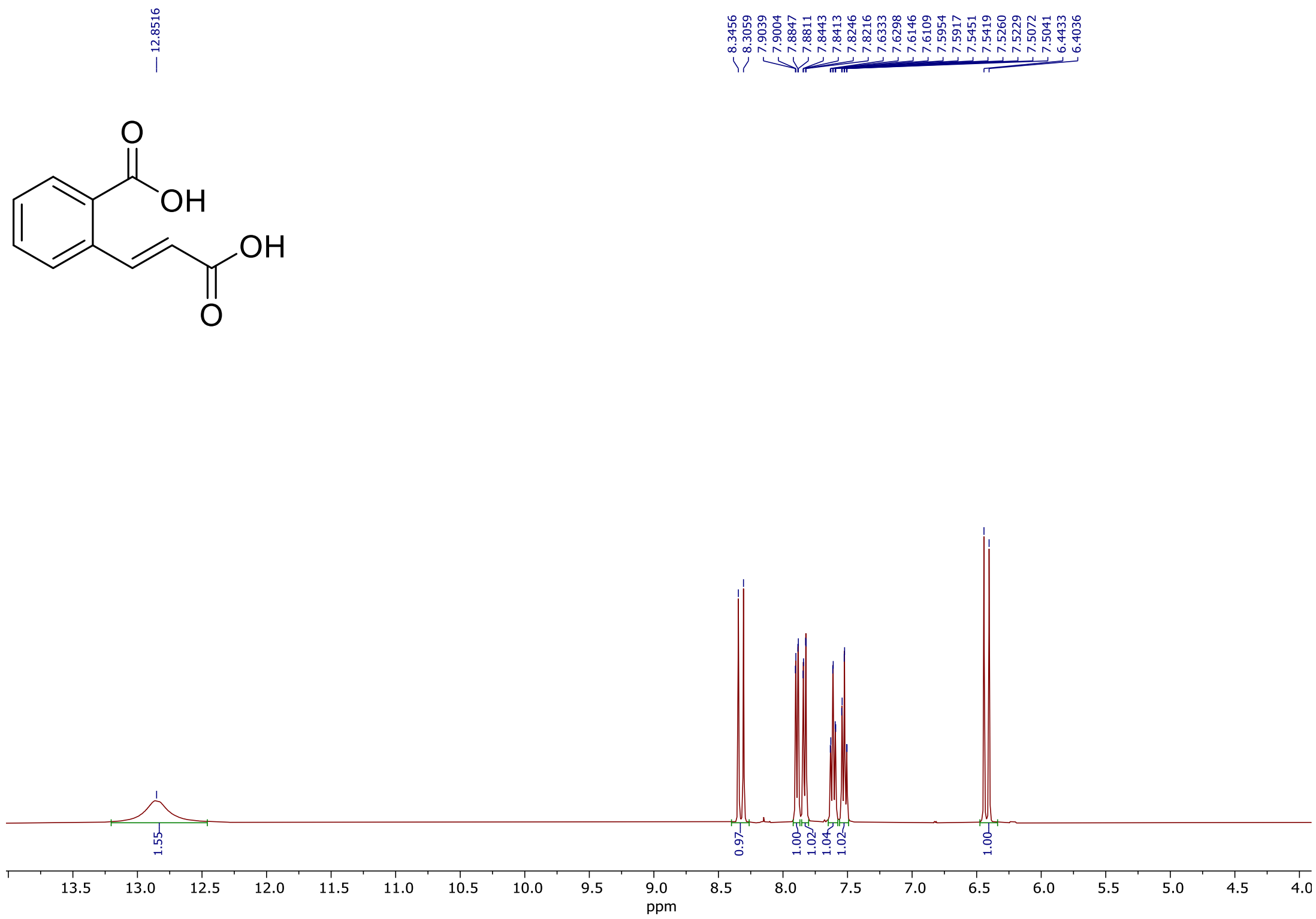

^{1}H NMR (400 MHz, DMSO-d_6); (*E*)-2-(2-Carboxyvinyl)benzoic acid **5**.

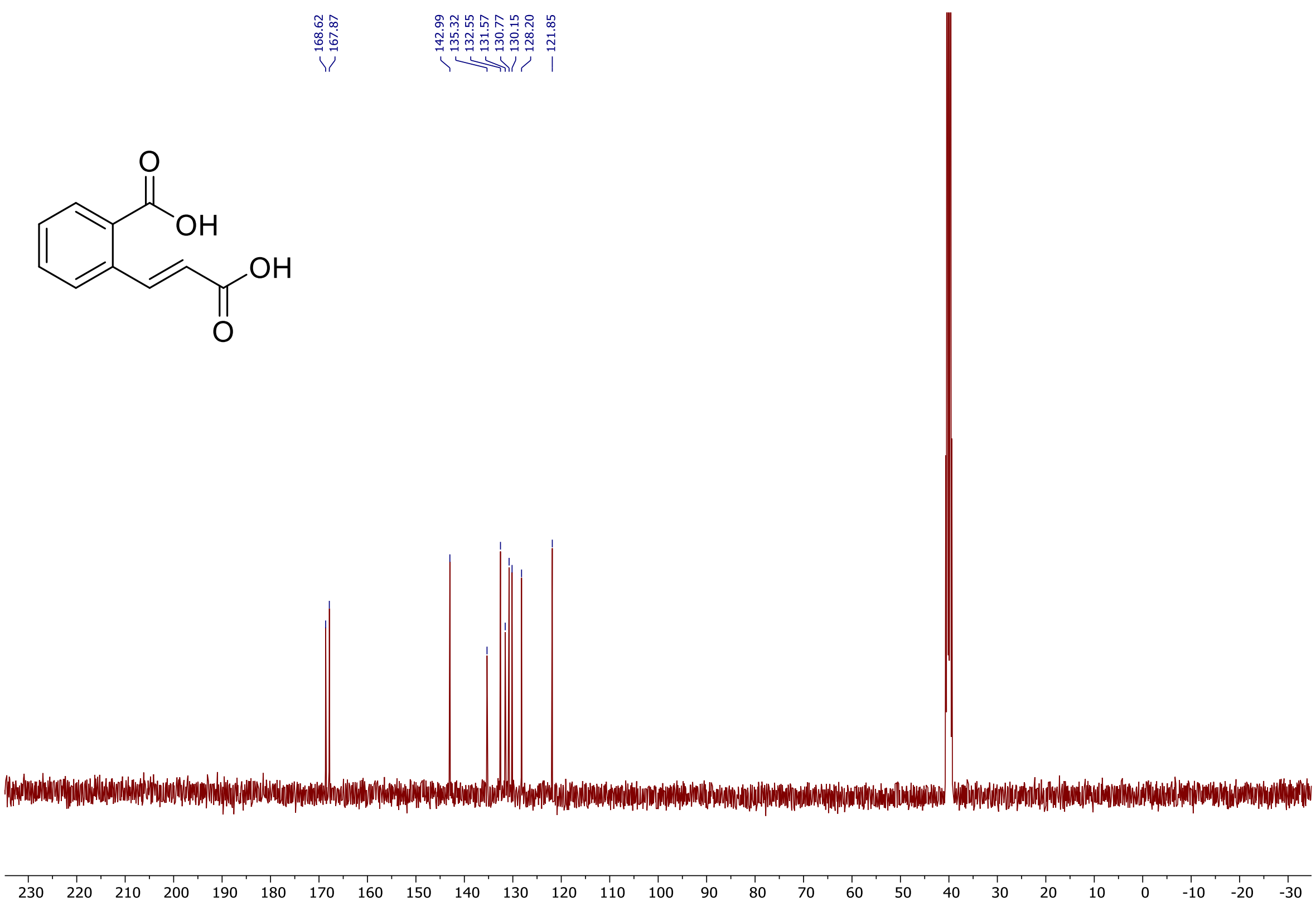

^{13}C NMR (100 MHz, DMSO-d_6); (*E*)-2-(2-Carboxyvinyl)benzoic acid **5**.

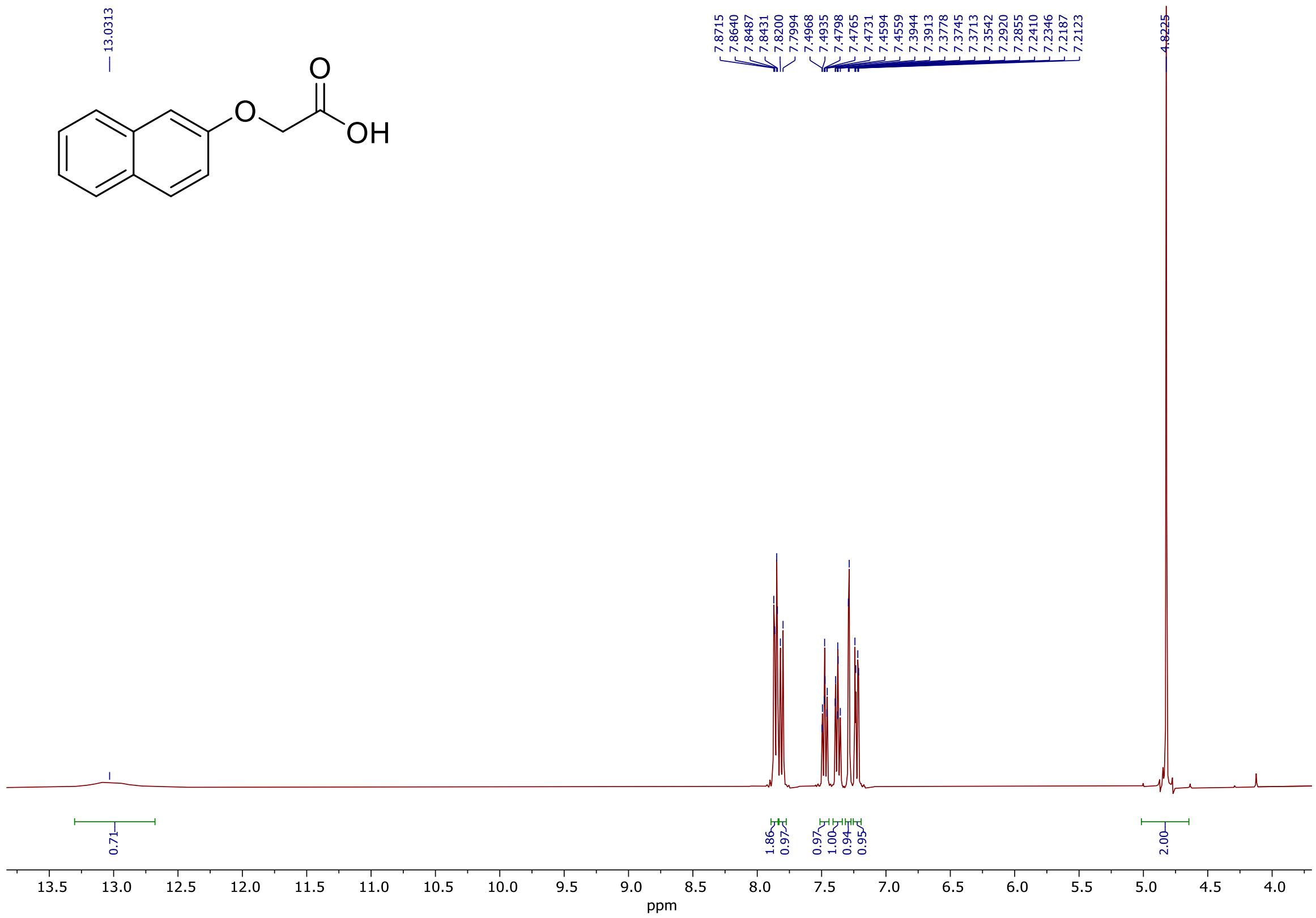

^{1}H NMR (400 MHz, DMSO-d_6); 2-(Naphthalen-2-yloxy)acetic acid **6**.

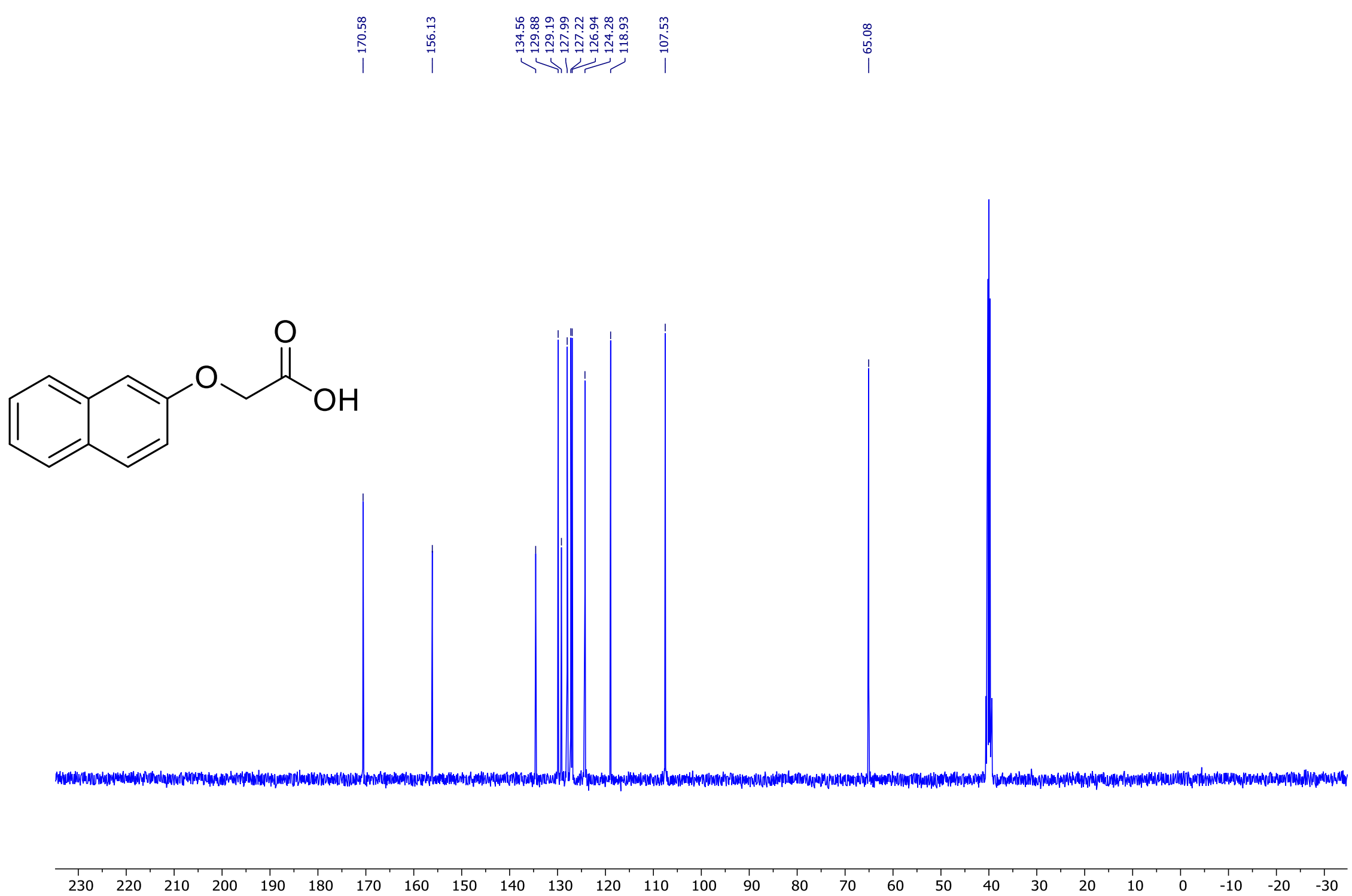

^{13}C NMR (100 MHz, DMSO-d_6); 2-(Naphthalen-2-yloxy)acetic acid **6**.
